# The role of *Plasmodium* V-ATPase in vacuolar physiology and antimalarial drug uptake

**DOI:** 10.1073/pnas.2306420120

**Published:** 2023-07-18

**Authors:** Arne Alder, Cecilia P. Sanchez, Matthew R. G. Russell, Lucy M. Collinson, Michael Lanzer, Michael J. Blackman, Tim-Wolf Gilberger, Joachim M. Matz

**Affiliations:** ^a^Cell Biology of Human Parasites Group, Centre for Structural Systems Biology, Hamburg 22607, Germany; ^b^Cellular Parasitology Department, Bernhard Nocht Institute for Tropical Medicine, Hamburg 20359, Germany; ^c^Department of Biology, University of Hamburg, Hamburg 20146, Germany; ^d^Center of Infectious Diseases, Parasitology, Heidelberg University Hospital, Heidelberg 69120, Germany; ^e^Electron Microscopy Science Technology Platform, The Francis Crick Institute, London NW1 1AT, United Kingdom; ^f^Centre for Ultrastructural Imaging, King’s College London, London SE1 1UL, United Kingdom; ^g^Malaria Biochemistry Laboratory, The Francis Crick Institute, London NW1 1AT, United Kingdom; ^h^Faculty of Infectious and Tropical Diseases, London School of Hygiene & Tropical Medicine, London WC1E 7HT, United Kingdom

**Keywords:** malaria, V-ATPase, vacuole, chloroquine, *Plasmodium falciparum*

## Abstract

To ensure their survival in the human bloodstream, malaria parasites degrade up to 80% of the host erythrocyte hemoglobin in an acidified digestive vacuole. Here, we combine conditional reverse genetics and quantitative imaging approaches to demonstrate that the human malaria pathogen *Plasmodium falciparum* employs a heteromultimeric V-ATPase complex to acidify the digestive vacuole matrix, which is essential for intravacuolar hemoglobin release, heme detoxification, and parasite survival. We reveal an additional function of the membrane-embedded V-ATPase subunits in regulating morphogenesis of the digestive vacuole independent of proton translocation. We further show that intravacuolar accumulation of antimalarial chemotherapeutics is surprisingly resilient to severe deacidification of the vacuole and that modulation of V-ATPase activity does not affect parasite sensitivity toward these drugs.

## Significance

Malaria is caused by *Plasmodium* parasites, which replicate within human erythrocytes. During intraerythrocytic growth, the parasite takes up and digests host cell hemoglobin in an acidified vacuole. Several antimalarials interfere with biochemical pathways in this vacuole leading to parasite death. It was long believed that accumulation of these drugs is a mere function of the pH gradient between parasite cytosol and the vacuolar lumen. By targeting the multimeric proton pump powering this gradient, we identify distinct functions of this protein complex in physiology and maintenance of the digestive vacuole. Contrary to current belief, we find that severe dissipation of the proton gradient has only limited impact on antimalarial drug uptake and no effect on drug susceptibility.

Malaria is caused by infection of red blood cells (RBCs) with parasites of the genus *Plasmodium*. These unicellular eukaryotes grow and replicate within an intraerythrocytic parasitophorous vacuole until they egress and invade new RBCs (1). Throughout intraerythrocytic development, the malaria parasite endocytoses host cell cytoplasm at the cytostomes, prominent invaginations of the parasite plasma membrane and the parasitophorous vacuole membrane that feature an electron-dense collar around the narrow opening (2, 3). Double-membrane vesicles are thought to pinch off from the cytostomal invaginations in order to traffic to the parasite’s digestive vacuole (DV), an acidified compartment in which up to 80% of the host cell cytoplasm is degraded over the course of one intraerythrocytic developmental cycle (3, 4). How these double-membrane vesicles fuse with the single membrane of the DV and how this results in intravacuolar hemoglobin release are unclear.

Inside the DV, hemoglobin is degraded by a number of proteases, facilitating amino acid acquisition and ensuring osmotic stability of the infected RBC (5, 6). Hemoglobin catabolism in the DV also releases the cofactor heme, which is promptly oxidized to hematin. Owing to its inherent cytotoxicity, the parasite mineralizes the hematin to bioinert hemozoin crystals, which accumulate in the DV over the course of intraerythrocytic maturation (7, 8). Hemoglobin digestion and the biomineralization of hematin are essential for parasite survival (6, 9). Importantly, hemozoin formation is the proven target of several antimalarial drugs, most notably the 4-aminoquinolines (4-AQ), which interfere with crystal growth and thereby cause hematin-induced oxidative damage (9, 10). In addition, the current front-line artemisinin-based antimalarials are activated by heme originating from hemoglobin catabolism and can also inhibit hematin biomineralization (11, 12).

The DV of *Plasmodium falciparum*, the most virulent human malaria parasite, is acidified to a pH of ~5 (13). As in other degradative compartments, this is believed to facilitate the unfolding of proteins to make them more accessible to proteolytic cleavage. Accordingly, hemoglobin degradation by DV proteases is most active around pH 5 (14). In vitro crystallization assays have suggested that hemozoin formation might also be pH dependent (15). Importantly, it has been proposed that the transvacuolar pH gradient may also drive the uptake of 4-AQ antimalarials, such as chloroquine (CQ). CQ accumulates up to 1,000-fold in the DV, which has been attributed to its weak base properties (16, 17). The CQ free base is thought to be membrane permeable. Once inside the acidic DV, CQ would gain a positive charge through protonation, thereby trapping and concentrating the drug (18). Thus, maintenance of an acidic pH in the parasite DV is likely essential for hemoglobin-to-hemozoin transition, parasite survival, and antimalarial drug uptake.

**Fig. 1.**
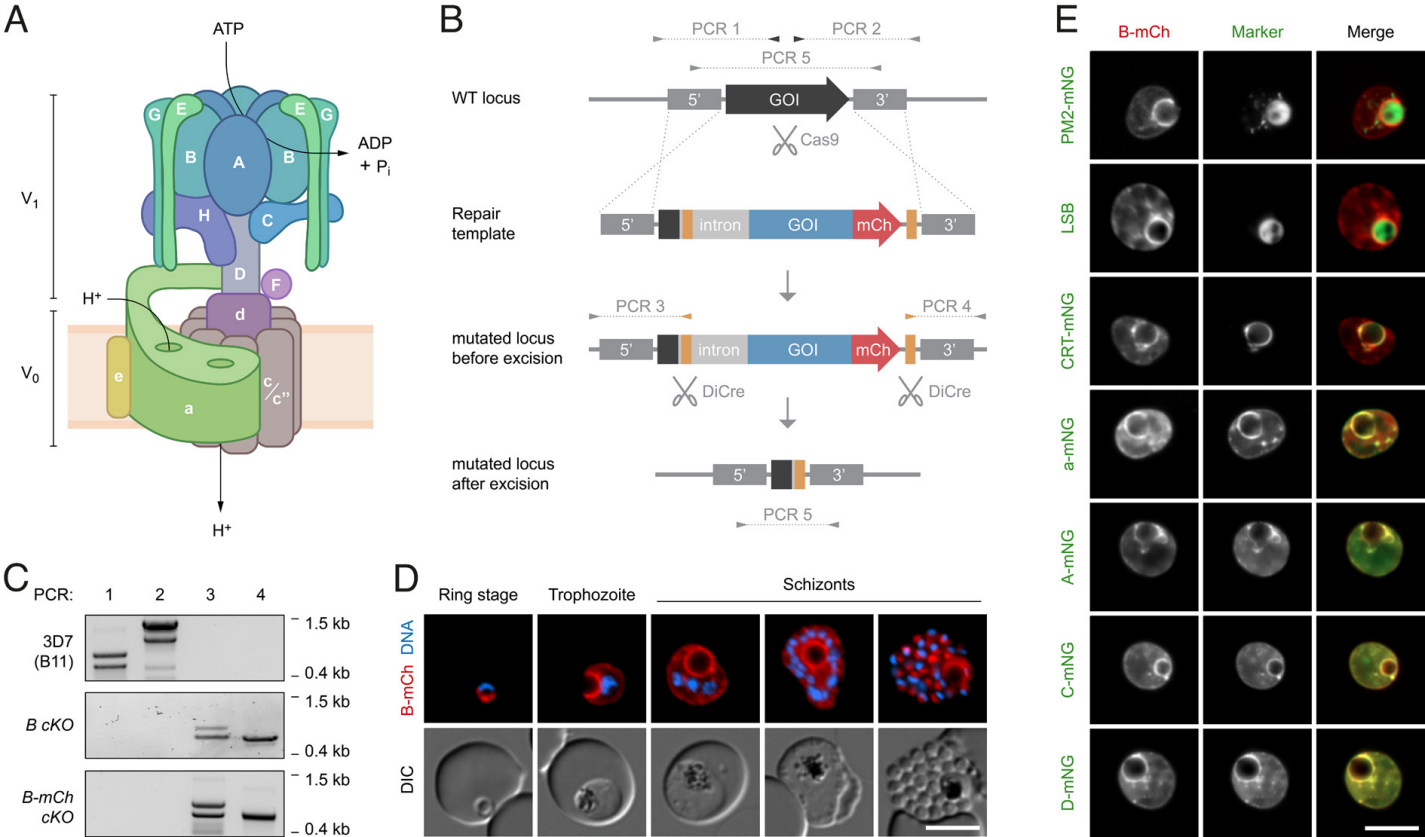
Expression and subcellular localization of *P. falciparum* V-ATPase. (*A*) Schematic representation of the eukaryotic V-ATPase. Subunits of the V_1_ and the V_0_ sector are denoted with uppercase and lowercase letters, respectively. ATP hydrolysis at the V_1_ sector energizes transmembrane proton transport at the V_0_ sector. (*B*) Genetic strategy for tagging and conditional deletion of V-ATPase subunits. Cas9-mediated double-strand cleavage of the gene of interest (GOI) and double homologous integration of a synthetic repair template yield transgenic parasites expressing the GOI in-frame with mCh. In some templates, mCh was omitted. *loxP* sites (yellow) are situated in an artificial intron and downstream of the coding sequence. RAP-induced dimerization of DiCre recombinase results in genomic excision of the *loxP*-flanked sequence. Primers used in diagnostic PCRs and expected amplicons are denoted with arrows and dotted lines. (*C*) Validation of conditional subunit B knockout parasites. Primers indicated in (*B*) were used in diagnostic PCRs to analyze genomic DNA from the parental B11 strain and from nontagged and mCh-tagged conditional knockout mutants. Note that the occurrence of secondary bands was independent of different primer combinations, PCR protocols, and DNA preparations, perhaps indicating occasional polymerase slippage at repetitive sequence stretches. (*D*) Expression and localization of subunit B throughout intraerythrocytic development, as observed by live fluorescence microscopy of *B-mCh cKO* parasites. DNA, Hoechst 33342. (*E*) Subunit B accumulates at the DVM and colocalizes with other V-ATPase subunits. *B-mCh cKO* parasites were stained with Lysosensor Blue DND-167 (LSB) or genetically engineered to express endogenous mNG-tagged proteins of interest. Shown are representative live fluorescence micrographs. A, C, D, and a: V-ATPase subunits; CRT: chloroquine resistance transporter; PM2: plasmepsin 2. (Scale bars, 5 µm.)

In eukaryotic organisms, organellar acidification is facilitated by a highly conserved heteromultimeric proton pump called vacuolar-type adenosine triphosphatase (V-ATPase). V-ATPase is a membrane-associated protein complex that couples ATP hydrolysis at a cytosolic V_1_ sector to the translocation of protons at a membrane-embedded V_0_ sector, thereby facilitating proton transport against a concentration gradient (19) (Fig. 1*A*). V_1_ consists of eight different subunits, each denoted with uppercase letters. This includes the catalytic hexamer formed by subunits A and B, which mediates ATP hydrolysis and causes rotation of a central stalk consisting of subunits D and F (20). Three peripheral stalks formed by subunits E and G hold the catalytic hexamer stationary (20). The V_0_ sector consists of five different subunits, denoted with lowercase letters. Subunit d couples the V_1_ rotary stalk to a membrane-embedded ring of proteolipid subunits, of which there can be different isoforms called c, c′, and c″ (20, 21). Rotation of this c-ring relays protons in cooperation with subunit a (20, 21). Many organisms express multiple isoforms of subunit a, enabling differential targeting and tissue-specific expression of different V-ATPase complex configurations (22). The molecular functions of the remaining subunits are less well defined, but they are important for the dynamic assembly of the complex and regulation of its activity (19). Apart from its core function in proton translocation, additional roles of V-ATPase have been identified in different organisms, including involvement in endocytosis, membrane fusion, nutrient and pH sensing, and signaling pathways (19).

The functions of V-ATPase in *Plasmodium* biology remain unknown. The parasite genome encodes all subunits required for the assembly of a V-ATPase complex, including a single isoform of subunit a and two isoforms of subunit c (c and c″), but the subcellular localization of the complex is unclear. Immuno-EM analysis has indicated that the catalytic hexamer localizes to the DV membrane (DVM), to intraparasitic vesicles, and to the parasite periphery (23), whereas immunofluorescence analysis suggested that subunit B is exported to the cytosol and plasma membrane of the infected RBC (24). Cell-based assays with the pH-sensitive dye fluorescein-dextran have revealed a proton pumping activity at the DVM that is stimulated by ATP and sensitive to glucose deprivation (25). Treatment with the established V-ATPase inhibitors bafilomycin A_1_ and concanamycin A (ConA) raised the vacuolar pH, together suggesting a function of *Plasmodium* V-ATPase in DV acidification (25). In addition, pharmacological V-ATPase inhibition impaired the parasite’s ability to acidify the external medium and to recover from experimentally induced cytosolic acidification, thus implicating a plasma membrane resident fraction of the complex in cytosolic pH homeostasis (26). Importantly, *Plasmodium* V-ATPase has been implicated in resistance toward potent antimalarial compounds currently under clinical development (27, 28).

To date, the biological role(s) of DV acidification and the underlying molecular machinery that controls the process remain largely unexplored. In this study, we combine reverse genetics with quantitative imaging, drug sensitivity, and transport assays to characterize the functions of *P. falciparum* V-ATPase in DV physiology and antimalarial drug susceptibility. We show that abrogation of V-ATPase-mediated proton pumping causes fatal deficiencies in intravacuolar hemoglobin release and heme sequestration and uncover a second function of the V_0_ sector in maintaining DVM stability independent of proton pumping. Importantly, we find that the uptake of CQ is unexpectedly resilient to inhibition of vacuolar proton transport and that parasite sensitivity toward 4-AQs cannot be modulated by reduced V-ATPase activity.

## Results

### Expression and Localization of *P. falciparum* V-ATPase during Asexual Blood Stage Development.

To study the function of the *Plasmodium* V-ATPase complex, we generated conditional knockout (*cKO*) parasites for V_1_ sector subunit B (PF3D7_0406100) on the genetic background of the dimerizable Cre recombinase (DiCre)-expressing B11 strain of *P. falciparum* (3D7) (29). Using Cas9 technology, two *loxP* sites were introduced into the subunit B locus, one within an artificial intron near the start codon and one downstream of the stop codon (Fig. 1*B*). These modifications were thus designed such that treatment of the transgenic parasites with exogenous rapamycin (RAP) would result in DiCre-mediated excision of 93% of the subunit B coding sequence, including the nucleotide-binding site required for ATP hydrolysis. Two independent conditional knockout lines were generated; in the *B-mCh cKO* line, subunit B was tagged with mCherry (mCh) at the C terminus, while in the *B cKO* line, the C terminus was left untagged. Site-specific integration of the modifying constructs into the parasite genome was confirmed by diagnostic PCR (Fig. 1*C*).

Live fluorescence microscopy of the *B-mCh cKO* mutant revealed abundant expression of subunit B throughout the asexual intraerythrocytic cycle, with localization predominantly to a rim around the hemozoin pigment (Fig. 1*D*). Live colocalization with the mNeonGreen (mNG)-tagged DV matrix protein plasmepsin 2 (PM2, PF3D7_1408000) and with the acidotropic dye Lysosensor Blue DND-167 confirmed this compartment as the acidic DV (Fig. 1*E*). Accordingly, a substantial fraction of subunit B colocalized with the mNG-tagged DVM protein chloroquine resistance transporter (CRT, PF3D7_0709000), indicating a DVM localization of the V-ATPase complex (Fig. 1*E*). In addition, a smaller fraction of subunit B localized to the parasite periphery and internal vesicular structures, a staining pattern which became more pronounced with progressing parasite maturation. Importantly, the localization of subunit B overlapped with mNG-tagged V-ATPase subunits A (PF3D7_1311900), C (PF3D7_0106100), D (PF3D7_1341900), and a (PF3D7_0806800), suggesting that the entire V-ATPase complex assembles at the DVM and rendering the possibility of tag-induced mislocalization negligible (Fig. 1*E*). None of the tagged V-ATPase subunits were observed in the cytoplasm or plasma membrane of the host erythrocyte, not supporting their putative export, as previously proposed (24).

### Subunit B Is Essential for V-ATPase Assembly and Parasite Blood Stage Survival.

**Fig. 2.**
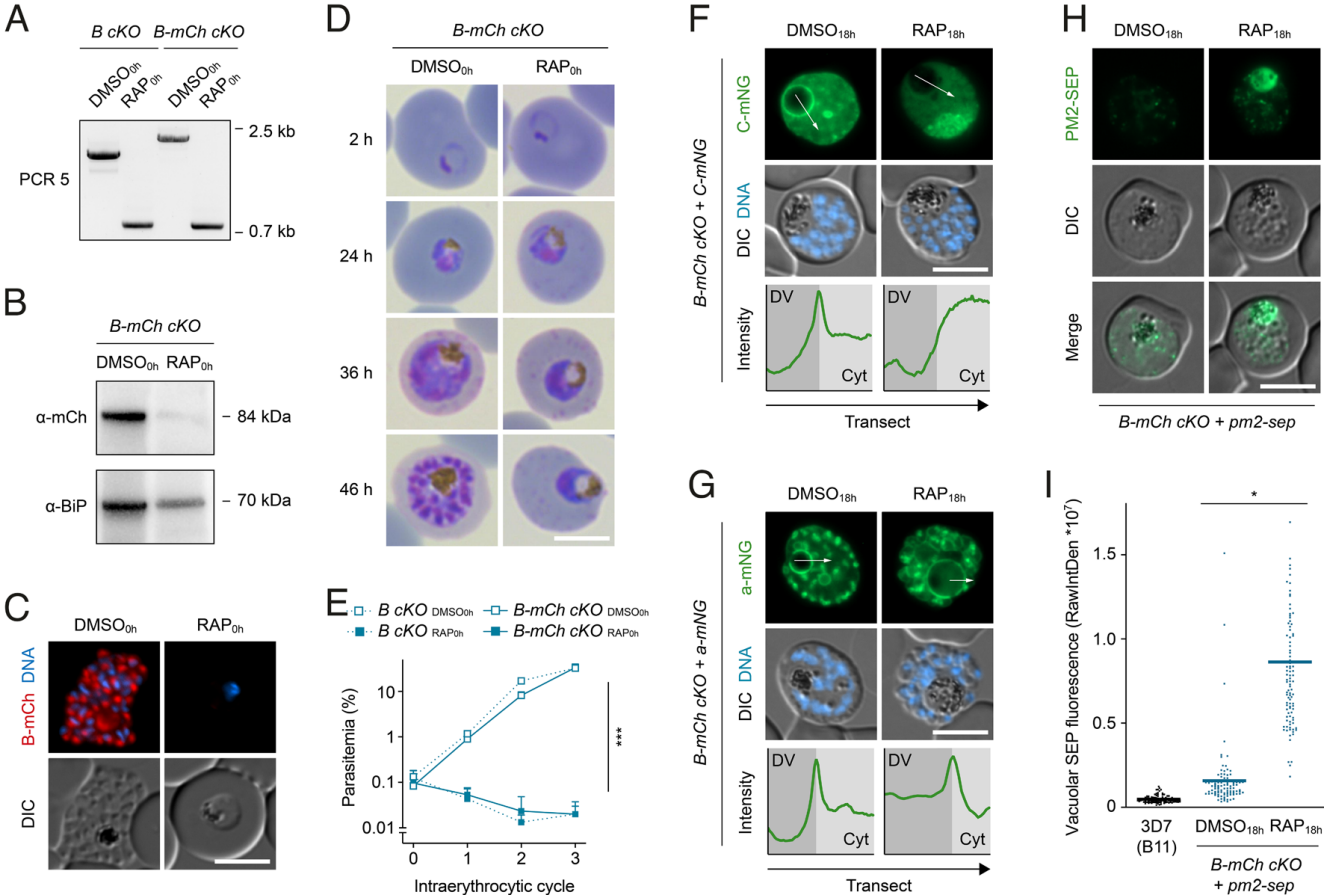
Loss of V-ATPase subunit B causes vacuolar deacidification and parasite death. (*A*) RAP-induced excision of *loxP*-flanked genomic DNA in *B cKO* and *B-mCh cKO* parasites. Parasites were treated with DMSO/RAP from the ring stage onward. Genomic DNA was harvested at the end of the cycle and subjected to diagnostic PCR5 as depicted in Fig. 1*B*. (*B* and *C*) RAP-induced loss of subunit B protein. *B-mCh cKO* parasites were treated as in (*A*). (*B*) Western blot of parasite extracts and (*C*) live fluorescence microscopy. DNA, Hoechst 33342. (*D* and *E*) Subunit B–deficient parasites arrest intraerythrocytic development. Shown are (*D*) Giemsa-stained parasites throughout the course of one cycle and (*E*) population growth dynamics over three cycles. Mean ± SD; two-way ANOVA; n = 3. (*F* and *G*) Loss of subunit B causes mislocalization of subunit C but not a. *B-mCh cKO* parasites expressing mNG-tagged subunits C (*F*) and a (*G*) were treated with DMSO/RAP from 18 h post invasion onward and imaged live at the schizont stage. Images are representative of >50 analyzed cells per condition. Transects (white arrows) spanning DV lumen and parasite cytoplasm (Cyt) were used to generate mNG intensity profiles. (*H* and *I*) The absence of subunit B causes DV deacidification. *B-mCh cKO* parasites expressing PM2 fused to SEP were treated and recorded as in (*F*). (*H*) Representative micrographs and (*I*) quantification of vacuolar SEP fluorescence as raw integrated density (RawIntDen). B11, control for autofluorescence. Individual and mean values (bars); paired *t* test; n = 99 parasites from three experiments. *P < 0.05; ***P < 0.001. (Scale bars, 5 µm.)

To study the effect of subunit B inactivation on parasite blood stage development, we induced DiCre-mediated genomic excision by treating tightly synchronized *B-mCh cKO* and *B cKO* parasites with RAP from the ring stage onward (RAP_0h_). Diagnostic PCR confirmed that the *loxP*-flanked sequence was efficiently excised from the parasite genome in both parasite lines (Fig. 2*A*). Accordingly, expression of the tagged protein in the *B-mCh cKO* line was substantially depleted at the end of the same intraerythrocytic cycle, as indicated by western blot analysis and live fluorescence microscopy (Fig. 2 *B* and *C*). Ablation of subunit B caused a complete halt in parasite maturation. While dimethyl sulfoxide (DMSO_0h_)-treated control parasites progressed normally through the ring, trophozoite, and schizont stages, development of the RAP_0h_-treated parasites arrested at the mononuclear trophozoite stage (Fig. 2*D*), resulting in a tight block in parasite replication (Fig. 2*E*). These observations lend additional support to our localization approach based on endogenous tagging since any interference of the fluorescent tags with V-ATPase function would have severely affected parasite fitness, which was not the case. We conclude that the parasite V-ATPase is essential for intraerythrocytic growth of *P. falciparum*.

We then examined whether deletion of subunit B affects assembly of the V-ATPase complex by studying the localization of mNG-tagged subunits A, C, D, and a in the *B-mCh cKO* line. To avoid observation of secondary effects of parasite mortality, we induced gene deletion 18 h after invasion (DMSO_18h_/RAP_18h_), which allowed parasite maturation and an appropriate comparison between viable parasites at the schizont stage (*SI Appendix*, Fig. S1). In DMSO_18h_-treated controls, subunits A, C, D, and a were abundantly expressed at the DVM. However, upon RAP_18h_-induced ablation of subunit B, subunits A, C, and D all mislocalized to the parasite cytosol, whereas the localization of subunit a remained unchanged (Fig. 2 *F* and *G* and *SI Appendix*, Fig. S2*A*). These results indicate that the V_1_ sector subunits are interdependent in their assembly and that deletion of subunit B causes inactivation of the entire V_1_ sector. By contrast, insertion of V_0_ sector subunits into the DVM appears to be independent of the V_1_ sector.

### Disturbed pH Homeostasis in V_1_-Deficient Parasites.

To establish the proton translocation activity of *Plasmodium* V-ATPase in cellulo, we introduced a pH-sensitive fluorescent biosensor into the DV of *B-mCh cKO* parasites by fusing to the C terminus of PM2 a GFP derivative called superecliptic pHluorin (SEP), fluorescence of which is efficiently quenched at acidic pH. We reasoned that RAP_18h_-induced V_1_ disassembly should abolish V-ATPase-mediated proton pumping and hence cause vacuolar deacidification, which would be detectable by increased SEP fluorescence. As shown in Fig. 2 *H* and *I*, this was indeed the case; in DMSO_18h_-treated control parasites, only a faint SEP signal was observed in the DV, and most of the fluorescence was associated with vesicular structures, likely representing nonacidified endosomal compartments en route to the DV (Fig. 2*H*). In contrast, RAP_18h_-induced deletion of subunit B caused a 7.5-fold increase of SEP fluorescence in the DV, fully consistent with a function of the *Plasmodium* V-ATPase in vacuolar proton translocation (Fig. 2 *H* and *I*).

Depletion of V-ATPase activity is also expected to affect the parasite’s cytosolic pH as a consequence of reduced proton transport across the DVM and the plasma membrane. Therefore, we determined the cytosolic pH of saponin-released *B cKO* parasites using the fluorescent pH indicator 2′,7′-bis-(2-carboxyethyl)-5-(and-6)-carboxyfluorescein acetoxymethyl ester (BCECF). At physiological external pH values, DMSO_18h_-treated parasites maintained a near-neutral cytosolic pH, whereas the cytoplasm of RAP_18h_-treated parasites appeared to be slightly more acidic, although this did not reach statistical significance (*SI Appendix*, Fig. S3*A*). Upon acidification of the external medium, both DMSO_18h_- and RAP_18h_-treated parasites responded with a reduction in cytosolic pH (*SI Appendix*, Fig. S3*A*). Importantly, the pH difference between DMSO_18h_- and RAP_18h_-treated *B cKO* parasites became more pronounced with decreasing pH of the medium, suggesting hypersensitivity of subunit B–deficient parasites toward external acidification (*SI Appendix*, Fig. S3 *A* and *B*). These data are in support of earlier findings implicating V-ATPase activity at the parasite plasma membrane in cytosolic pH homeostasis (26). Together, our findings suggest that V-ATPase translocates protons across the plasma membrane and the DVM of *P. falciparum* parasites.

### Defective Hemoglobin Release in the DV of V_1_-Deficient Parasites.

**Fig. 3.**
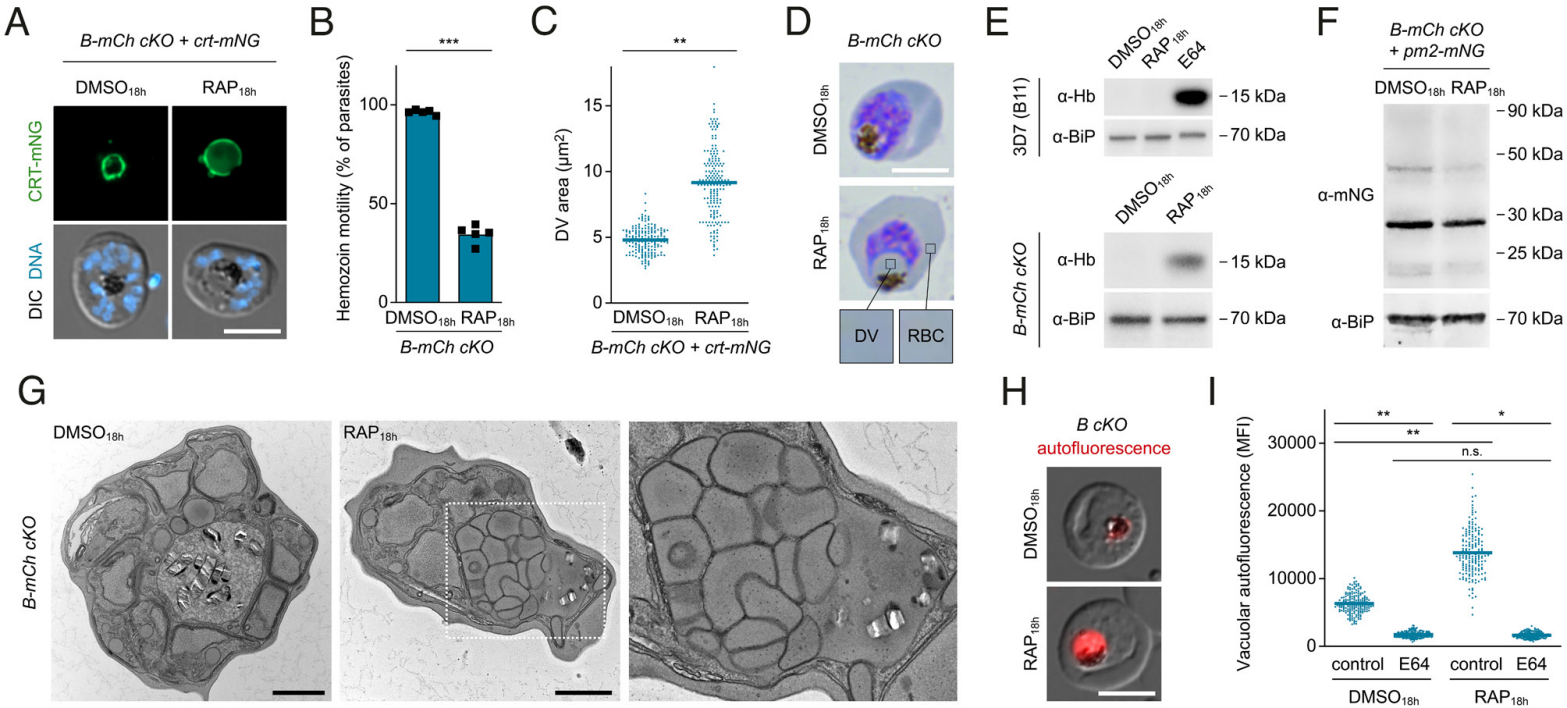
The absence of V-ATPase subunit B impairs intravacuolar hemoglobin release and heme sequestration. (*A*–*C*) DV swelling and reduced hemozoin motility upon loss of subunit B. *B-mCh cKO* parasites expressing CRT-mNG were treated with DMSO/RAP from 18 h post invasion onward and imaged live at the schizont stage. (*A*) Representative fluorescence micrographs. DNA, Hoechst 33342. (*B*) Fraction of parasites displaying hemozoin motility. n = 5. (*C*) CRT-mNG-delineated area. n = 180 parasites from three experiments. Individual and mean values (bars); paired *t* test. (*D* and *E*) The swollen DV of subunit B–deficient parasites contains native hemoglobin. *B-mCh cKO* parasites were (*D*) visualized by Giemsa staining or (*E*) released by saponin and subjected to western blot. Parental B11 parasites were further treated with protease inhibitor E64 (21.7 µM). Hb, hemoglobin α. (*F*) Western blot of *B-mCh cKO* parasites expressing PM2-mNG. The mNG signal is predominantly associated with a band of ~28 kDa, corresponding to mNG alone. Unprocessed PM2-mNG (~78 kDa) was not detected. (*G*) Transmission electron micrographs of *B-mCh cKO* parasites. Note the accumulation of intra-DV vesicles (zoomed region, dashed white frame). In 9% of DMSO_18h_- (n = 32) and 84% of RAP_18h_-treated parasites (n = 51), the hemozoin-containing DV matrix displayed an electron density comparable to that of the RBC cytoplasm. (*H* and *I*) Subunit B–deficient parasites display elevated porphyrin fluorescence, which depends on hemoglobin catabolism. *B cKO* parasites were treated as in (*E*) and imaged live. (*H*) Representative micrographs and (*I*) quantification of DV autofluorescence as mean fluorescence intensity (MFI). Individual and mean values (bars); one-way ANOVA and Tukey’s multiple comparisons test; n = 180 parasites from three experiments. n.s., nonsignificant; *P < 0.05; **P < 0.01; ***P < 0.001. All scale bars, 5 µm except panel *G* (1 µm).

During the above analysis, we noticed what appeared to be expansion of the DV upon loss of subunit B. To examine this in more detail, we studied the localization of mNG-tagged CRT in these parasites. This revealed a consistent morphological phenotype in the RAP_18h_-treated parasites, in which the DV was markedly swollen with the hemozoin crystals concentrated to one side of the vacuole (Fig. 3*A*). This was accompanied by a clear reduction in hemozoin crystal motility in live parasites (Fig. 3*B*). The area delineated by the CRT signal was increased by ~twofold in the subunit B–null parasites, which corresponds to an ~2.6-fold increase in volume, assuming spherical DV proportions (Fig. 3*C*). Examination of Giemsa-stained thin blood films showed that the coloration of the vacuolar contents of subunit B–null parasites was identical to that of the host cell compartment, suggesting the accumulation in the DV of undigested hemoglobin (Fig. 3*D*). To confirm this, parasites released from their host cells by saponin lysis were subjected to western blot analysis using antibodies specific for human hemoglobin α. As controls for these experiments, we included parental B11 parasites treated with DMSO_18h_, RAP_18h_, or with the cysteine protease inhibitor E64, which blocks proteolytic hemoglobin catabolism in the DV (30). As shown in Fig. 3*E*, E64 caused substantial accumulation of ingested hemoglobin in the control parasites, whereas DMSO_18h_ and RAP_18h_ had no apparent effect. Importantly, intraparasitic hemoglobin accumulation was also observed in RAP_18h_-treated *B-mCh cKO* parasites but not in DMSO_18h_-treated controls (Fig. 3*E*). To our surprise, accumulation of hemoglobin in subunit B–deficient parasites did not coincide with a lack of vacuolar proteolysis since we observed efficient cleavage of a PM2-mNG fusion protein upon treatment with either DMSO_18h_ or RAP_18h_ (Fig. 3*F*). Upon microscopic analysis of RAP_18h_-treated *B-mCh cKO* parasites, we noticed that a minor population of parasites displayed a heterogenous distribution of PM2-mNG with marked accumulations in subregions of the swollen DV (*SI Appendix*, Fig. S4).

Thus, to probe for the ultrastructural basis of these observations, we studied *B-mCh cKO* parasites by transmission electron microscopy. In DMSO_18h_-treated control parasites, the DV lumen appeared more electron lucent than the RBC cytoplasm and contained hemozoin crystals evenly distributed throughout the vacuolar matrix (Fig. 3*G*). By marked contrast, subunit B–null parasites displayed a bloated DV filled with single membrane vesicles, which displaced the hemozoin crystals toward the DVM, likely limiting their ability to twirl freely in live parasites (Fig. 3*G*). Notably, both the hemozoin-containing matrix and the lumen of the intravacuolar vesicles displayed an electron density comparable to that of the host cell cytosol. We conclude that in the absence of V-ATPase-mediated DV acidification, hemoglobin-containing vesicles delivered to the DV matrix fail to undergo disruption to release their contents. Our findings highlight the process of intravacuolar vesicle disruption and hemoglobin release as a key event required for parasite maturation and demonstrate a marked pH dependency of the underlying molecular machinery.

### Lack of V-ATPase Subunit B Increases Intravacuolar Porphyrin Fluorescence.

As hemoglobin catabolism in the DV releases heme, which is converted into hemozoin crystals, hemozoin formation can be used as surrogate to quantify hemoglobin digestion or trafficking. To examine this in the subunit B–null parasites, we used quantitative polarization microscopy to show that RAP_18h_-treated *B-mCh cKO* parasites formed only ~82% of the hemozoin quantities observed in controls, although this difference did not reach statistical significance under the tested conditions (*SI Appendix*, Fig. S5). We reasoned that an abnormal pH in the DV may also influence hemozoin formation in a more direct fashion, e.g., by altering the protonation state of hematin and thus hindering its incorporation into crystalline hemozoin. We therefore predicted that the DV of subunit B–deficient parasites would exhibit elevated concentrations of free heme species. To address this, we recorded and quantified the red autofluorescence of the DV, which is believed to originate from free heme-derived porphyrins (31). To validate this approach, we first tested whether the endogenous fluorescence of the DV was dependent upon active hemoglobin catabolism. Inhibition of hemoglobin digestion with E64 prevents the release of heme from globin chains (32). Accordingly, E64 treatment of parental B11 parasites was found to reduce vacuolar fluorescence by 65% (*SI Appendix*, Fig. S6). Conversely, we reasoned that an elevation of the heme progenitor protoporphyrin IX in the RBC cytoplasm should cause increased red fluorescence in the DV (31). Consistent with this, we indeed found that stimulation of protoporphyrin IX synthesis inside the host cell compartment by addition of 5-aminolevulinic acid leads to a significant increase in DV autofluorescence (*SI Appendix*, Fig. S5). These data suggest that endogenous vacuolar fluorescence can indeed be used as a proxy for the concentration of free heme-derived porphyrins in the *Plasmodium* DV.

Having validated our approach, we quantified DV autofluorescence in (non-tagged) *B cKO* parasites treated with either DMSO_18h_ or RAP_18h_. Strikingly, RAP_18h_ treatment caused a 2.2-fold elevation of DV autofluorescence compared to DMSO_18h_-treated controls (Fig. 3 *H* and *I*). Treatment with E64 antagonized this RAP_18h_-dependent increase in autofluorescence (Fig. 3*I*). Importantly, DV autofluorescence of E64-treated subunit B-null parasites was comparable to that of E64-treated parental B11 parasites (Fig. 3*I* and *SI Appendix*, Fig. S6*B*), suggesting that DV deacidification alone does not affect autofluorescence in the absence of hemoglobin catabolism. Combined, these results indicate a role of V-ATPase-mediated DV acidification in enabling efficient hematin biomineralization in *Plasmodium* parasites.

### The V_0_ Sector of V-ATPase Regulates DVM Morphology Independent of Proton Translocation.

**Fig. 4.**
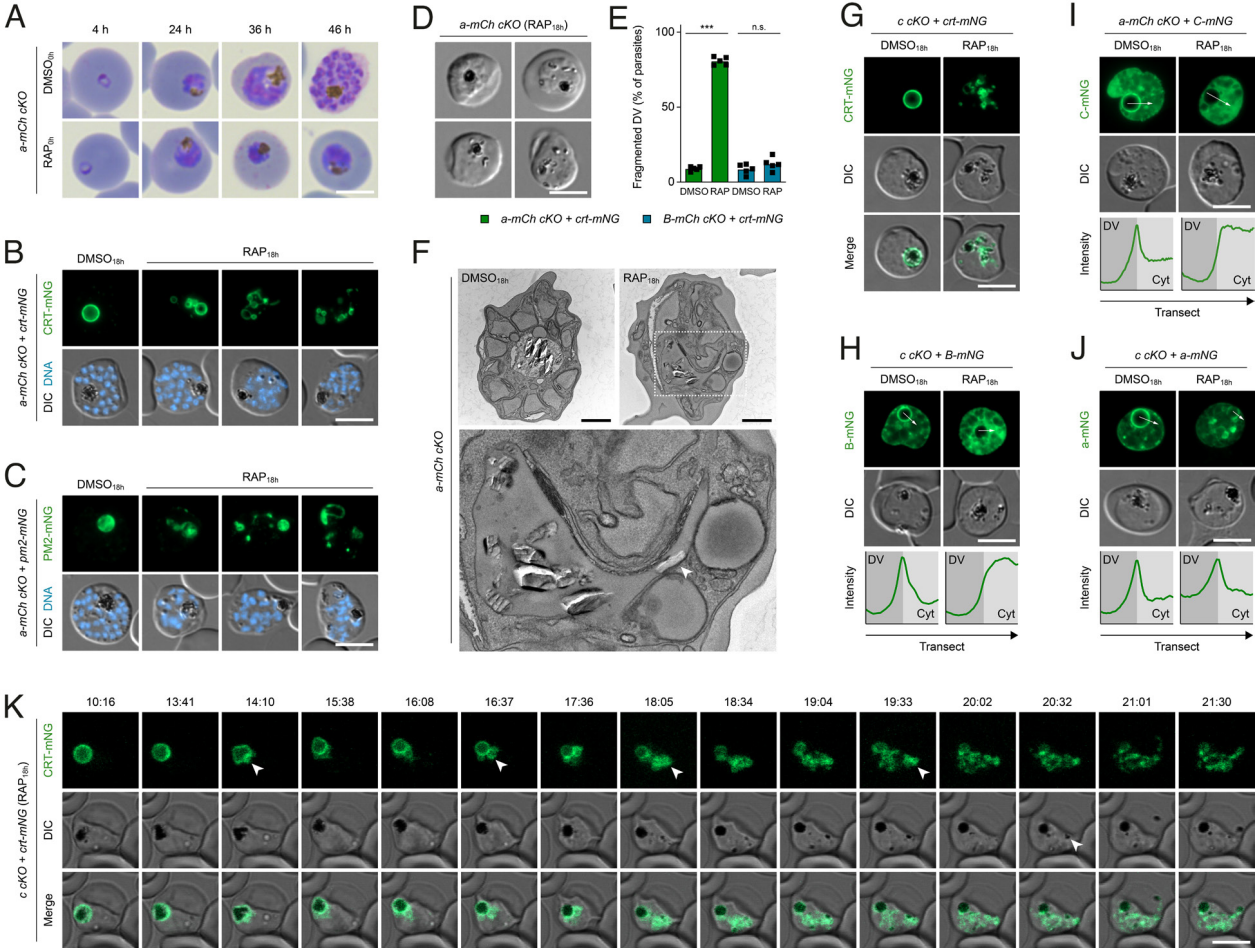
The V-ATPase V_0_ sector stabilizes the DVM independent of proton translocation. (*A*) Subunit a–deficient parasites arrest intraerythrocytic development. *a-mCh cKO* parasites were treated with DMSO/RAP from the ring stage onward and visualized by Giemsa staining throughout the course of one cycle. (*B* and *C*) DVM fragmentation upon loss of subunit a. *a-mCh cKO* parasites expressing CRT-mNG (*B*) or PM2-mNG (*C*) were treated with DMSO/RAP from 18 h post invasion onward and imaged live at the schizont stage. DNA, Hoechst 33342. (*D*) DIC images of subunit a–deficient parasites showing different degrees of hemozoin scattering. (*E*) Quantification of DV fragmentation in *a-mCh cKO* and *B-mCh cKO* parasites expressing CRT-mNG. Individual and mean values (bars); n.s., nonsignificant; ***P < 0.001; paired *t* test; n = 5. (*F*) Transmission electron micrographs of *a-mCh cKO* parasites. Dashed white frame, zoomed region. Arrowhead, hemozoin in tubular DVM feature. (*G*) DVM fragmentation upon loss of subunit c. *c cKO* parasites expressing CRT-mNG were treated and imaged as in *B*. (*H* and *I*) Deletions of subunits a and c cause V_1_ disassembly. *c cKO* (*H*) and *a-mCh cKO* parasites (*I*) expressing mNG-tagged subunits B or C, respectively, were treated as in *B* and imaged live. Transects (white arrows) spanning DV lumen and parasite cytoplasm (Cyt) were used to generate mNG intensity profiles. (*J*) DVM localization of subunit a in subunit c–deficient parasites. (*K*) Confocal time-lapse microscopy of a RAP_18h_-treated *c cKO + crt-mNG* parasite. Average CRT-mNG intensity projections alongside a single DIC z-section. Numbers, elapsed time since first recording (hours:minutes). Arrowheads from left to right: DVM collapsing; focal DVM accumulation; DVM protruding; DVM fragment; mislocalized hemozoin. All scale bars, 5 µm except panel *F* (1 µm).

We next sought to determine whether inactivation of a V_0_ sector subunit would recapitulate the defects observed upon deletion of subunit B. For this, using the same genetic strategy, we generated conditional knockout parasites of subunit a, a membrane-embedded V-ATPase component with crucial functions in proton relay. After confirming successful modification of the locus and the RAP-induced loss of DNA and protein (*SI Appendix*, Fig. S7 *A* and *D*), we studied the effects of subunit a inactivation on parasite development. Mirroring our observations with subunit B, ablation of subunit a from the ring stage onward caused a loss of parasite proliferation resulting from developmental arrest at the trophozoite stage (Fig. 4*A* and *SI Appendix*, Fig. S7*E*). Strikingly, we found that the absence of subunit a did not cause swelling of the DV but its fragmentation into numerous CRT-mNG-positive compartments (Fig. 4*B*). This did not coincide with permeabilization of the DVM, as indicated by the localization of PM2-mNG (Fig. 4*C*), but was frequently accompanied by scattering of the hemozoin crystals throughout the parasite (Fig. 4*D*). Importantly, DV fragmentation was not observed upon deletion of subunit B (Fig. 4*E*). Ultrastructural inspection of subunit a–deficient parasites by transmission electron microscopy revealed a DVM with tubular and vesicular features collapsing around the hemozoin crystals (Fig. 4*F*). As described above for subunit B, RAP induction of all subunit a mutants was commenced 18 h post invasion to ensure parasite viability (*SI Appendix*, Fig. S7 *F* and *G*).

To determine whether subunit a is the only V-ATPase subunit regulating DVM architecture or whether this function is shared by other V_0_ sector subunits, we generated a conditional knockout mutant of subunit c (*c cKO*, PF3D7_0519200; *SI Appendix*, Fig. S8 *A* and *B*) and studied its DV morphology. Disruption of subunit c caused abrogation of intraerythrocytic development (*SI Appendix*, Fig. S8 *C* and *D*), which was accompanied by DV fragmentation, reproducing the phenotype of subunit a-null parasites (Fig. 4*G*). Disruptions of subunits a and c caused disassembly of the V_1_ sector, as observed by live fluorescence microscopy of mNG-tagged subunits A, B, and C (Fig. 4 *H* and *I* and *SI Appendix*, Fig. S2*B*). By contrast, deletion of subunit c did not affect the membrane localization of subunit a (Fig. 4*J*). Together, these observations indicate that V-ATPase-mediated control of DVM morphology is not facilitated by subunit a alone but requires an assembled V_0_ sector. The fact that subunit B inactivation causes deacidification but not fragmentation of the DV also suggests that the proton transport activity of the V-ATPase complex is not required to maintain an intact DV. This concurs with our observation that the V_1_ sector disassembles in all three of the V-ATPase subunit knockouts regardless of which sector is affected.

The appearance of the DV in subunit c–null parasites was further analyzed by time-lapse microscopy to clarify whether vacuolar fragmentation results from fission of the DVM or from impaired fusion of the DVM with pre-DV vesicles. We found that the DVM of these parasites collapsed around the hemozoin crystals, which was followed by agglomeration of DVM material at one side of the vacuole (Fig. 4*K* and Movie S1). These membrane accumulations then extended into the parasite cytoplasm and disintegrated into multiple separate compartments in a process that took 2 to 3 h. Combined, these observations indicate that disruption of the V_0_ sector induces fission of the preexisting DVM.

### Unaltered Sensitivity of V_1_-Deficient Parasites toward Hemozoin-Targeting Antimalarials.

**Fig. 5.**
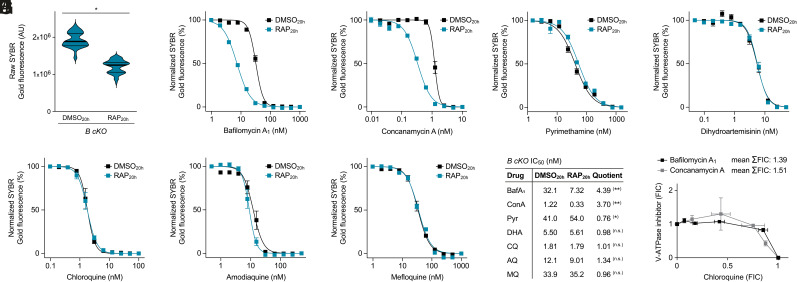
V-ATPase activity does not modulate parasite susceptibility toward DV-targeted antimalarials. (*A*) Delayed induction of subunit B deletion allows completion of the intraerythrocytic cycle and reinvasion. Ring-stage cultures were inoculated at 1% parasitemia and treated with DMSO/RAP from 20 h post invasion onward. Shown is the SYBR Gold fluorescence of synchronized *B cKO* cultures 72 h post invasion. Noninfected red blood cell cultures were used for background correction. Violin plots including median (fat bars) and quartile values (thin bars); paired *t* test; n = 63 cultures from three experiments. (*B*–*H*) Dose–response curves of *B cKO* parasites in the presence of varying concentrations of (*B*) bafilomycin A_1_ (BafA_1_), (*C*) concanamycin A (ConA), (*D*) pyrimethamine (Pyr), (*E*) dihydroartemisinin (DHA), (*F*) chloroquine (CQ), (*G*) amodiaquine (AQ), or (*H*) mefloquine (MQ) supplied 20 h post invasion. Parasites were treated and DNA quantified as described in *A*. Fluorescence was normalized to the lowest non-inhibitory drug concentrations. Mean ± SEM; n = 3. (*I*) IC_50_ values inferred from the dose–response curves in (*B*–*H*). The fold-change in sensitivity is expressed as the quotient of IC_50_ values from DMSO_20h_- and RAP_20h_-treated parasites. Paired *t* test; n = 3. (*J*) Fixed ratio isobologram analysis of the interactions of CQ with BafA_1_ and ConA. FIC values were obtained from serial dilutions of 5:0, 4:1, 3:2, 2:3, 1:4, and 0:5 drug ratios. Mean ± SEM; n = 3. n.s., nonsignificant; *P < 0.05; **P < 0.01.

It is currently believed that the pH gradient across the DVM is the driving force behind the accumulation of CQ and other 4-AQ antimalarials (18). If this were the case, we predicted that severe reduction of V-ATPase activity, as observed in subunit B-null parasites, should diminish vacuolar drug uptake and cause reduced 4-AQ sensitivity. To address this, we induced gene deletion in the *B cKO* mutant 20 h post invasion (DMSO_20h_/RAP_20h_), which allowed the parasites to complete intraerythrocytic development and transition into a second cycle, albeit at reduced efficiency (Fig. 5*A*). Using this protocol, we then measured reinvasion in the presence of varying drug concentrations supplied at trophozoite stage (20 h post invasion). To validate our approach, we first tested the susceptibility of *B cKO* parasites toward the V-ATPase inhibitors bafilomycin A_1_ and ConA. As expected, we observed hypersensitivity toward these compounds in RAP_20h_-treated *B cKO* parasites as compared to DMSO_20h_ controls, with IC_50_ values being roughly four times lower (Fig. 5 *B* and *C*). This was not due to a general fragility of subunit B–deficient parasites toward chemotherapeutic insult since sensitivities toward the antifolate pyrimethamine and the endoperoxide dihydroartemisinin were not affected by loss of subunit B (Fig. 5 *D* and *E*). Surprisingly, using the same experimental setup, we found that parasite susceptibility toward the 4-AQs CQ and amodiaquine remained unchanged in subunit B–deficient parasites (Fig. 5 *F*, *G*, and *I*). The same was true for the related quinolinemethanol mefloquine (Fig. 5 *H* and *I*). To account for the rapid action of some of the tested antimalarials, we repeated these assays with the adaptation that compounds were added to the parasites at the young schizont stage (36 h post invasion). This again revealed unaltered sensitivity of subunit B–deficient parasites toward all tested antimalarials with the exception of the two V-ATPase inhibitors (*SI Appendix*, Fig. S9). To further characterize the relationship between V-ATPase activity and 4-AQ susceptibility, we performed fixed ratio isobologram analysis with parental B11 parasites, probing for the interactions between CQ and the V-ATPase inhibitors bafilomycin A_1_ and ConA, respectively. We found no evidence for synergistic or antagonistic interactions between CQ and these compounds, as all fractional inhibitory concentration values (FIC) fell in the range of additivity (Fig. 5*J*). Together, these findings seem to suggest that vacuolar deacidification in the sublethal but growth-inhibitory range does not impact susceptibility of *P. falciparum* parasites toward DV-targeted antimalarials.

### Impact of Vacuolar Deacidification on Antimalarial Drug Uptake.

**Fig. 6.**
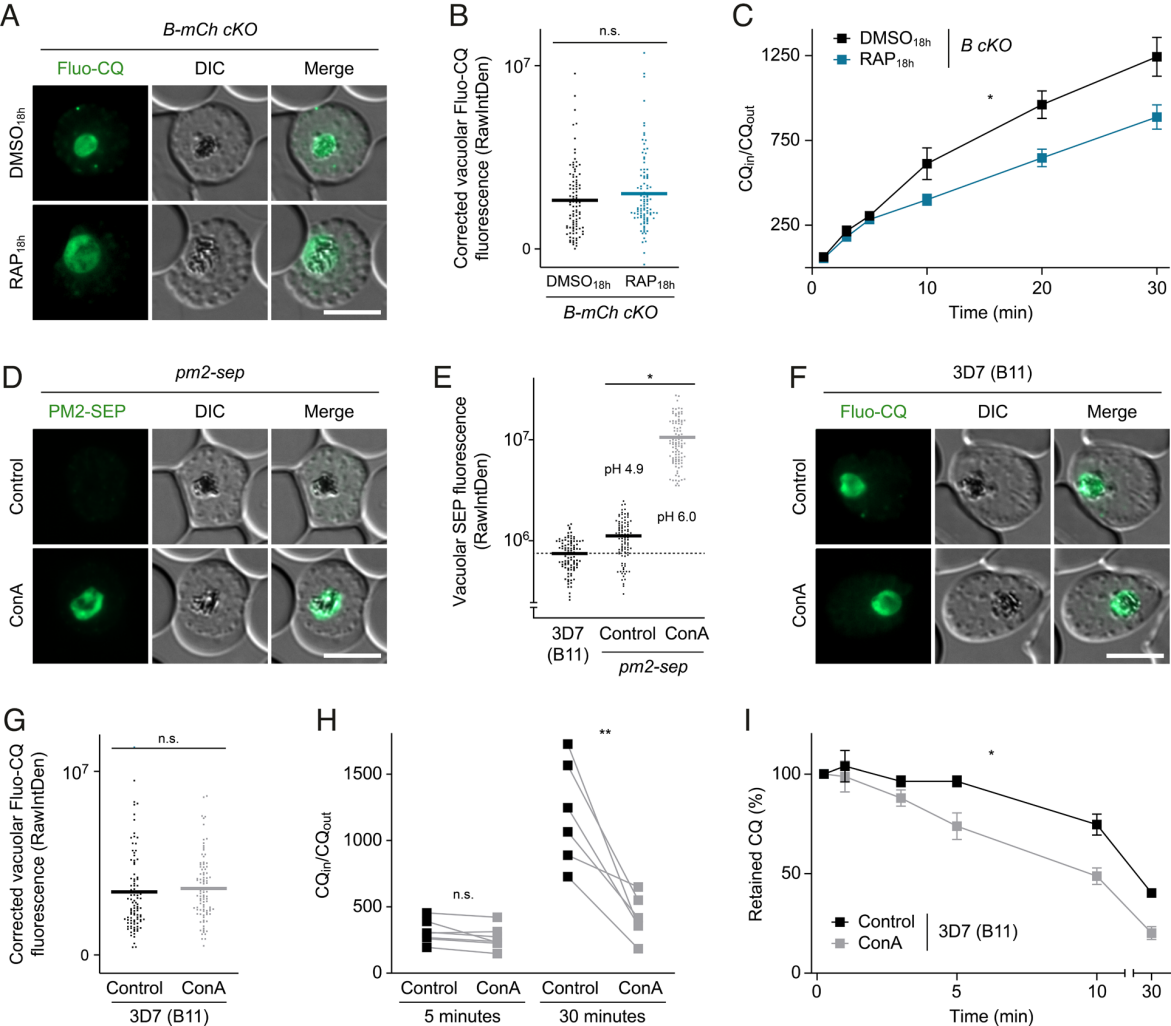
Impact of DV deacidification on intravacuolar drug accumulation. (*A* and *B*) Normal Fluo-CQ import in subunit B–deficient parasites. (*A*) Live fluorescence micrographs of DMSO_18h_- and RAP_18h_-treated *B-mCh cKO* parasites at the schizont stage incubated in the presence of 500 nM Fluo-CQ. (*B*) Quantification of vacuolar Fluo-CQ fluorescence as raw integrated density (RawIntDen). Individual and mean values (bars), corrected for mean intensity of unstained controls; paired *t* test; n = 99 parasites from three experiments. (*C*) [^3^H]CQ uptake by *B cKO* parasites. Schizont-infected RBCs were incubated in the presence of [^3^H]CQ and the ratio of intracellular versus extracellular [^3^H]CQ was determined over time. Mean ± SEM; Two-way ANOVA; n ≥ 4. (*D* and *E*) Pharmacological V-ATPase inhibition deacidifies the DV matrix. *pm2-sep* reporter parasites at the schizont stage were treated with 61 nM concanamycin A (ConA) or vehicle only for 30 min. (*D*) Live fluorescence micrographs and (*E*) quantification of vacuolar SEP fluorescence. B11, control for autofluorescence (dashed line). pH estimates obtained through calibration are indicated (*SI Appendix*, Fig. S10). Individual and mean values (bars); paired *t* test; n = 99 parasites from three experiments. (*F* and *G*) Live fluorescence micrographs (*F*) and quantification (*G*) of vacuolar Fluo-CQ fluorescence in the DV of B11 parasites treated with 61 nM ConA or vehicle only for 30 min. Individual and mean values (bars); paired *t* test; n = 99 parasites from three experiments. (*H* and *I*) Impaired accumulation and accelerated efflux of [^3^H]CQ by ConA-treated B11 parasites. (*H*) Drug accumulation was determined after 5 and 30 min as in (*C*). ConA or vehicle was added together with [^3^H]CQ at the start of the experiment. Connected data points, paired observations; paired *t* test; n ≥ 6. (*I*) Infected RBCs preloaded for 15 min with [^3^H]CQ were incubated in the presence of 61 nM ConA or vehicle only. Intracellular drug concentrations were then determined over time and normalized to initial values. Mean ± SEM; two-way ANOVA; n ≥ 3. n.s., nonsignificant; *P < 0.05; **P < 0.01. (Scale bars, 5 µm.)

These findings seemed to indicate that parasites with a deacidified DV can still efficiently take up hemozoin-targeting antimalarials. To test this, we quantified the import of the fluorescent CQ analog Fluo-CQ into the DV of *B-mCh cKO* parasites (33). Using quantitative live fluorescence microscopy, we found no difference in intravacuolar staining intensity between DMSO_18h_- and RAP_18h_-treated parasites (Fig. 6 *A* and *B*). Since Fluo-CQ fluorescence may be affected by factors unrelated to the pharmacology of the underivatized drug, we also measured the uptake of radiolabeled CQ ([^3^H]CQ) into the *B cKO* mutant. This revealed slightly reduced [^3^H]CQ uptake in subunit B–deficient parasites. After 30 min, RAP_18h_-treated parasites had accumulated 71% of the [^3^H]CQ levels observed in DMSO_18h_ controls (Fig. 6*C*).

To achieve a more complete deacidification of the DV, we incubated parasites with ConA at 50x IC_50_ for 30 min, which increased vacuolar SEP fluorescence >26-fold in our *pm2-sep* reporter line (Fig. 6 *D* and *E*). By calibrating the sensor with the protonophore nigericin and media of varying pH, we determined that this change in fluorescence corresponds to a rise in vacuolar pH by approximately one pH unit, from 4.9 to 6.0 (Fig. 6*E* and *SI Appendix*, Fig. S10), which is predicted by the weak base trapping model to decrease CQ accumulation >150-fold (34). By contrast, we found that ConA treatment neither affects intravacuolar fluorescence of Fluo-CQ nor the initial stages of [^3^H]CQ accumulation in B11 parasites (Fig. 6 *F* and *H*). Incubation with ConA eventually resulted in a threefold reduction of accumulated [^3^H]CQ after 30 min (Fig. 6*H*). Similarly, continued ConA treatment caused B11 parasites to lose preloaded [^3^H]CQ slightly faster than untreated parasites, leading to a twofold reduction in retained radiolabel after 30 min (Fig. 6*I*). In light of the substantial DV deacidification induced by ConA, these changes in intravacuolar drug accumulation and retention fall far below the predictions of a simple weak base trapping model and heavily imply a more sophisticated drug partitioning behavior and/or the involvement of alternative uptake routes.

## Discussion

In this work, we undertook a reverse genetic characterization of the *Plasmodium* V-ATPase and identified essential functions of this complex in DV physiology. We show that V-ATPase activity drives acidification of the parasite DV, which is required for intravacuolar hemoglobin release. Two scenarios of how hemoglobin might be released from double-membrane vesicles into the DV are conceivable: 1) either the inner membrane is lysed inside the transport vesicle and the resultant single membrane vesicle fuses with the DV or 2) the outer vesicle membrane fuses with the DVM to release a single membrane vesicle into the DV matrix. Our finding of intravacuolar vesicle accumulation in subunit B–null parasites is in strong support of the second scenario and agrees with rare observations of hemoglobin-filled vesicles in the DV of parasites treated with CQ or with the actin-depolymerizing agent cytochalasin D (35, 36). Importantly, the fatal deficits in hemoglobin release in our subunit B mutants clearly demonstrate the essential role of vacuolar acidification for parasite survival during the blood stage and also suggest that the previously reported activity of a proton-pumping V-type pyrophosphatase at the DVM (25) cannot compensate for the loss of V-ATPase function.

Our results further indicate a pronounced pH sensitivity of the molecular machinery that facilitates membrane lysis inside the DV. The protein factors involved in this process could be lipases and/or pore-forming proteins. There have been no reports of a DV-localized lipase in malaria parasites thus far, but a hemolysin III homolog has been identified expressed inside the DV of *P. falciparum* (37). Recombinant hemolysin III was shown to induce lysis of human erythrocytes and *Xenopus laevis* oocytes through formation of a ~3.2-nm pore (37). Ectopic expression of GFP-tagged hemolysin III caused DV swelling in *P. falciparum*, but in contrast to the subunit B–null parasites, the DV matrix of this mutant was translucent suggesting efficient hemoglobin processing (37). A whole genome screen in *Plasmodium berghei* further suggests that hemolysin III is dispensable for asexual parasite proliferation in the blood (38). Thus, a potential involvement of this pore-forming protein in intravacuolar vesicle disruption remains to be investigated with reverse genetic approaches.

The blockade in hemoglobin release we observed made it challenging to analyze downstream deficits in hemoglobin proteolysis or hemozoin formation upon loss of subunit B. Nonetheless, despite efficient PM2-mNG processing, transmission electron microscopy indicated accumulation of undigested hemoglobin inside the hemozoin-containing DV matrix of subunit B–null parasites. This is in agreement with the reported low pH optimum of the DV proteases (14). In addition to the accumulation of undigested hemoglobin, we detected elevated porphyrin fluorescence in the DV of subunit B–deficient parasites, which points to defective heme sequestration. It was previously shown that hematin crystallization in acetate solution is pH dependent, indicating that only monoprotonated hematin participates in the mineralization process (15). A pH-induced shift in the equilibrium of non-, mono-, and diprotonated hematin species could thus explain the accumulation of non-sequestered porphyrins upon loss of subunit B. Accordingly, we observed a trend toward reduced hemozoin levels in subunit B–null parasites. We argue that the limited extent of this reduction results from our experimental setup, which enforces analysis of viable and identically sized parasites despite the essentiality of subunit B, together leading to an underestimation of the biological impact. A reduction in hemozoin quantities can be explained as a consequence of impaired hemoglobin release from intravacuolar vesicles. Nonetheless, our control experiments with E64 showed that lack of hemoglobin processing reduces DV autofluorescence rather than increasing it. We thus conclude that elevated porphyrin fluorescence is an additional effect of subunit B inactivation linked to the sequestration of heme.

Importantly, we identified a second function of the *Plasmodium* V-ATPase in controlling DVM morphology. Previous research has shown that V-ATPase regulates the fusion–fission equilibrium of vacuoles, lysosomes, and other acidified compartments, including the plant-like vacuole of the related apicomplexan parasite *Toxoplasma gondii* (39). However, the underlying mechanisms remain controversial. One school of thought suggests that organellar acidification is required for this function. This is supported by assays of homotypic vacuole fusion in yeast, in which expression of a pyrophosphate-driven monomeric proton pump from *Arabidopsis thaliana* rescued the fusion deficits resulting from loss of V-ATPase (40). By contrast, time-lapse imaging of live yeast cells suggests that vacuole fusion is actually induced, not repressed, upon chemical V-ATPase inhibition (41). Other studies find that acidification is not involved in fusion at all. A mutant version of the type III effector protein *Vibrio* outer protein Q (VopQ) from *Vibrio parahaemolyticus* completely collapses the proton gradient across V-ATPase-containing membranes without affecting fusion (42). Furthermore, the proteolipid components of V_0_ can be mutagenized in a fashion that impairs fusion but not acidification (43). Thus, concepts have emerged in which membrane fusion is promoted by one V_0_ sector or by interaction of two opposing V_0_ sectors in *trans* and in cooperation with additional fusion mediators such as Ras-related in brain (Rab) proteins and soluble *N*-ethylmaleimide-sensitive-factor attachment receptors (SNARE) (43, 44).

Our observations in live malaria parasites support a function of V-ATPase in DV morphogenesis that is independent of its proton pumping activity and the V_1_ sector. Ultrastructural analysis and time-lapse microscopy indicate that the loss of membrane-embedded V-ATPase subunits induces fission of the preexisting DV. A recent study used time-lapse microscopy to demonstrate that the DVM of *P. falciparum* undergoes fission to form small autonomous membrane lobes, which usually remain attached to the DV surface and which disappear minutes later, probably due to refusion with the mother DV (45). Although the biological function of this fission-fusion cycle remains enigmatic, it is conceivable that the disruption of the V_0_ sector interferes with this process. Together, our observations suggest that the V_0_ sector of the *Plasmodium* V-ATPase is an essential structural constituent of the vacuolar membrane that safeguards the integrity of the DV.

By targeting the parasite V-ATPase, we were able to test a longstanding theory about 4-AQ uptake by malaria parasites. It is currently believed that the accumulation of CQ and related hemozoin targeting antimalarials is driven by the pH difference between cytosol and DV matrix (17, 18). This has also led to the idea that resistance toward CQ can be gained by limiting drug uptake through minor alterations of the resting pH of the DV (46). This theory, initially proposed in 1972 (18), was tentatively confirmed by analyzing drug sensitivity and CQ uptake in response to manipulation of the external pH (46, 47). However, this treatment likely affects various variables, such as the protonation equilibrium of CQ and the activity of transporters at the parasite surface. It appears unlikely that external acidification can modulate the proton gradient across the DVM without substantially affecting cytosolic pH and thereby parasite viability. The use of broadly active lysosomotropic agents, such as ammonium chloride, appeared to decrease CQ uptake and susceptibility (46). However, the specificity of these treatments and their impact on parasite fitness remain unknown.

Our experimental approach circumvents these limitations by specifically targeting the machinery responsible for DV acidification through genetic and pharmacological inactivation of V-ATPase-mediated proton pumping, and we validate the resultant deacidification of the DV matrix by noninvasive in situ pH approximation. The use of a genetically encoded fluorescent pH biosensor fused to an endogenous DV protein allows precise spatial resolution and avoids artifacts resulting from exogenous fluorochrome loading which have previously caused large variations in DV pH estimates (34). Using this approach, we determined a vacuolar resting pH of 4.9, which falls within the range of previously reported values (13). Despite our efforts to ensure parasite viability at the schizont stage, slight differences in maturity between DMSO_18h_- and RAP_18h_-treated *B-mCh cKO + pm2-sep* parasites might have influenced the outcome in our reverse genetic experiments to a certain extent, as our pH readout is dependent on the expression of PM2. By contrast, this is of no concern for pharmacological V-ATPase inhibition, given the narrow treatment window.

Delayed RAP induction of the conditional subunit B deletion mutants allowed us to deacidify the DV matrix to the maximum extent still compatible with parasite survival. Although we detected in these parasites a mild deficit in [^3^H]CQ accumulation at the later stages of our time-course analysis, unaltered drug susceptibility seems to suggest that deacidification of the DV is not a functional pathway to gain resistance toward CQ and related antimalarials. We induced more severe deacidification with high concentrations of ConA, raising the DV pH by approximately one pH unit. This did not affect vacuolar labeling with the fluorescent CQ analog Fluo-CQ. Just as CQ, Fluo-CQ accumulates in the DV, binds hematin with high affinity, and is effluxed from the DV of CQ-resistant parasites by the action of CRT (33). However, the fluorescent nitrobenzofurazan moiety of Fluo-CQ might introduce unwanted pharmacological properties that deviate from CQ, perhaps altering membrane permeability and binding affinity for intracellular ligands. In addition, Fluo-CQ fluorescence may not accurately reflect accumulated quantities of the probe since various physicochemical factors within the DV might influence signal intensities. To address these potential shortcomings, we also measured the uptake and retention of underivatized CQ, which appeared to be affected upon prolonged ConA treatment. However, the amount of cell-associated CQ in these parasites was 50 to 80 times higher than predicted by the weak base trapping model, revealing a surprising resilience of vacuolar drug accumulation toward substantial DV deacidification.

It is challenging to define the physiological state of V-ATPase-deficient parasites in its entirety, and secondary effects of reduced parasite fitness might influence the observed phenotypes. However, any such bias would be expected to add to the specific deficiencies in drug accumulation, suggesting that the reductions in [^3^H]CQ uptake and retention that we observed might in fact be overestimates. Indeed, it was previously shown for saponin-released parasites that ConA initiates DV deacidification almost instantaneously (48), and we detected no differences in the early [^3^H]CQ uptake phase under the influence of ConA. Whether physiologically relevant or not, the reductions in [^3^H]CQ accumulation and retention that we detected upon prolonged ConA exposure fall far below the values predicted by the weak base trapping model. Our data defy a simple partitioning scenario in which only the CQ free base is membrane permeable and are more in agreement with a recent model, based on experimentally determined distribution coefficients for the non-, mono-, and di-protonated CQ species in a liposomal system, in which all CQ species can interact with lipid bilayers and eventually pass, albeit to different extent (49). According to this revised partitioning model, a shift in pH from 4.9 to 6.0, as seen in this study upon inactivation of V-ATPase, would reduce CQ entrapment only marginally (49).

The limited impact of vacuolar deacidification on drug import observed in this study might also hint at the existence of alternative uptake routes, such as active molecular transport or intracellular retention by hematin binding (50, 51). It was recently shown that CQ requires a nutrient-permeable channel in the parasitophorous vacuole membrane to reach the parasite (52), which is in direct conflict with the weak base trapping model. In support of this, CQ uptake was previously shown to be saturable, temperature dependent, and sensitive to the deprivation of glucose and ATP, which are all signatures of active molecular transport (33, 53, 54). One attractive candidate that might facilitate 4-AQ uptake into the DV is the P-glycoprotein homolog 1, also known as multidrug resistance protein 1 (MDR1). Apart from the V-ATPase complex, this ABC transporter is the only known DVM protein to transport its substrates into the DV (55). 4-AQ transport was observed in *X. laevis* oocytes upon heterologous MDR1 expression (56, 57), and mutations in the MDR1 coding sequence modulate 4-AQ susceptibility in allelic exchange experiments and in the field (58–61). *P. falciparum* MDR1 seems to share the broad substrate specificity of its mammalian ortholog toward cationic and neutral amphiphiles (56), which renders MDR1 an attractive candidate for vacuolar 4-AQ import. Combined, our observations strongly suggest that V-ATPase-mediated proton translocation cannot modulate parasite susceptibility toward hemozoin targeting antimalarials.

## Materials and Methods

### Parasite Cultivation.

*P. falciparum* blood stage parasites were grown in deidentified human RBCs (B+) and maintained in Roswell Park Memorial Institute medium 1640 (RPMI 1640) containing 0.5% AlbuMAXII (Thermo Fisher Scientific) at 37 °C in an atmosphere of 1% O_2_, 5% CO_2_, and 94% N_2_. For parasite synchronization, mature schizonts were enriched by Percoll density gradient centrifugation and added to fresh RBCs. Invasion was allowed to take place for 2 h under shaking conditions. The remaining schizonts were removed with a second Percoll gradient followed by sorbitol lysis. For reverse genetic experiments, these synchronized cultures were treated with 20 nM RAP or DMSO from 18 h post invasion onward and analyzed at the mature schizont stage, unless stated otherwise. This protocol avoids observation of secondary effects of parasite mortality, as it allows efficient parasite maturation but still reduces levels of the respective V-ATPase subunits down to 21% (subunit a) or 24% (subunit B) in RAP_18h_-treated parasites by the end of the cycle (*SI Appendix*, Figs. S1 and S7 *F* and *G*). For some experiments, 21.7 µM E64 or 10 µM 5-aminolevulinic acid was added 24 h post invasion.

### Generation and Validation of Transgenic Parasites.

Transgenic parasites were generated using established Cas9-mediated techniques, as described in *SI Appendix*.

### Assays of Parasite Replication and Drug Sensitivity.

Growth assays were initiated with DMSO_0h_- and RAP_0h_-treated ring-stage parasites and set up as described previously (62). Parasitemia was measured in two-day intervals on a FACSVerse (BD Biosciences) or NovoCyte (Acea Biosciences) flow cytometer, using SYBR Green nuclear dye (1:10,000; Thermo Fisher Scientific) to identify infected RBCs. Drug sensitivity assays were performed as described in *SI Appendix*.

### Light and Fluorescence Microscopy.

Giemsa-stained culture smears were imaged on a DM2000 LED light microscope (Leica) equipped with a DMC2900 camera (Leica). Fluorescence microscopy was performed with an Eclipse Ni light microscope (Nikon) fitted with a C11440 camera (Hamamatsu) or with a D6B fluorescence microscope (Leica) equipped with a DFC9000 GT camera (Leica). For detailed microscopy protocols including quantitative analysis of Fluo-CQ uptake, please refer to *SI Appendix*.

### Transmission Electron Microscopy.

Infected RBCs were Percoll-purified 42 h post invasion, before being fixed in 2.5% glutaraldehyde and 4% formaldehyde in 0.1 M phosphate buffer. After fixation, cells were embedded in 2% agarose and processed as described previously (29). In short, blocks were stained with reduced osmium and tannic acid, dehydrated with a graded ethanol and acetone series, and infiltrated with Epon resin (Embed 812, TAAB). Eighty-nanometer sections of the blocks were stained with lead citrate and imaged in a 1,400 FLASH transmission electron microscope (JEOL).

### Cytosolic pH Approximation.

The cytosolic pH of saponin-released parasites was approximated using the acetoxymethyl ester of BCECF, as described in *SI Appendix*.

### Chloroquine Response Assays.

Drug accumulation assays were performed with radiolabeled CQ as described in *SI Appendix*.

### Software and Statistical Analysis.

Fluorescence microscopy images were analyzed with FIJI (ImageJ2, version 2.3.0/1.53f). GraphPad Prism (version 9.0.1) was used for data visualization and statistical tests. For most experiments, DMSO- and RAP-treated parasites originating from the same cultures were analyzed with a paired two-tailed *t* test. Parasite growth assays and time-resolved [^3^H]CQ uptake and efflux assays were analyzed with a two-way ANOVA. Comparisons between more than two independent experimental groups were performed with a one-way ANOVA followed by Tukey’s multiple comparison test. For drug sensitivity assays, data were first normalized to the lowest noninhibitory concentration, and then, IC_50_ values were determined by nonlinear regression assuming variable slopes. Statistic tests were performed with mean values from independent experiments and not with data points that originate from individual parasites or technical replicates. Final figures were compiled with Adobe Illustrator (version 25.2.1).

## Supplementary Material

Appendix 01 (PDF)

Dataset S01 (XLSX)

Movie S1. Time-lapse microscopy reveals DVM fission upon loss of V-ATPase subunit c. *c cKO* parasites expressing CRT-mNG were tightly synchronized, treated with RAP from 18 hours post invasion onward and imaged live on a confocal microscope throughout intraerythrocytic development. For each timepoint, average CRT-mNG intensity projections from 3D reconstructions are shown alongside a single z-section of DIC. Time stamp, hours:minutes.

## Data Availability

All study data are included in the article and/or supporting information.
