# Supplementary material for: The role of Plasmodium V-ATPase in vacuolar physiology and antimalarial drug uptake
Source: Proc Natl Acad Sci U S A. 2023 Jul 18;120(30):e2306420120. doi: 10.1073/pnas.2306420120 (PMC10372686; doi:10.1073/pnas.2306420120)
Supplement: Supplementary file 1 — Appendix 01 (PDF) [file pnas.2306420120.sapp.pdf]

PNAS

Supporting Information for

The role of *Plasmodium* V-ATPase in vacuolar physiology and antimalarial drug uptake

Arne Alder, Cecilia P. Sanchez, Matthew R. G. Russell, Lucy M. Collinson, Michael Lanzer, Michael J. Blackman, Tim-Wolf Gilberger, Joachim M. Matz

Joachim M. Matz
Email: joachim.matz@bnitm.de

This PDF file includes:

Supporting text
Figures S1 to S11
Tables S1 and S2
Sequences S1 to S13
Legend for Movie S1
Legend for Dataset S1
SI References

Other supporting materials for this manuscript include the following:

Movie S1
Dataset S1

Supporting text – Methods

Generation and validation of transgenic parasites

P. falciparum B11 parasites constitutively expressing DiCre were co-transfected with a pDC2 guide plasmid inducing Cas9-mediated double strand cleavage of the locus of interest, together with a linearized repair template (1). Templates were generated by commercial gene synthesis and contained 5′ and 3′ homology regions and a recodonized sequence of the gene. In case of the conditional knockout mutants, two *loxP* sites were introduced into the sequence, one within an artificial intron and one downstream of the stop codon. Tagging with fluorescent proteins was achieved by cloning mCh, mNG or SEP into the repair template in frame with the coding sequence, using HpaI and EcoRV restriction sites. Transgenic parasites were selected with WR99210 and cloned by limiting dilution. Isolation of transgenic parasite clones and DiCre-mediated genomic excision were confirmed by diagnostic PCR and fluorescence microscopy. Primers for molecular cloning, guide plasmid construction and diagnostic PCR as well as repair template sequences are included in Fig. 1*B* and *SI Appendix*, Table S1 and Sequences S1 to 13. Transgenic parasite strains generated in this study are listed in *SI Appendix*, Table S2.

Drug sensitivity assays

Drug sensitivity assays were performed as described previously (2). In brief, synchronous *B cKO* parasite cultures were seeded into 96 well plates 20 hours post invasion at a final volume of 200 µl per well at 2% hematocrit and 1% parasitemia in the presence of RAP (20 nM) or DMSO, respectively. Antimalarial compounds at varying concentrations were added in triplicate 20 or 36 hours post invasion. Plates were incubated for a total of 72 hours to allow for parasite reinvasion to occur. Following cell lysis, parasite DNA was labelled with 1x SYBR Gold stain (Invitrogen) and fluorescence was quantified using an EnVision multilabel plate reader (PerkinElmer, 485 nm excitation, 535 nm emission. Uninfected RBCs served as a background control (for raw data, see Dataset S1). For drug interaction studies, CQ was mixed with the V-ATPase inhibitors at fixed ratios of 5:0, 4:1, 3:2, 2:3, 1:4, and 0:5. The drug combinations were then diluted and used in drug sensitivity assays as described above. Mean ∑FIC values were calculated and interpreted as described previously (3, 4).

Quantitative live fluorescence microscopy

For quantification of protein expression, DV autofluorescence, vacuolar morphology and V-ATPase subunit dissociation, parasites were recorded 42 hours post invasion. For quantification of heme-related autofluorescence, the red signal of the DV of non-tagged parasites was captured using a standard Texas Red filter cube configuration. Vacuolar fluorescence intensity and area were determined after manual outlining of the DV. To determine levels of V-ATPase subunit expression, the signal was captured from the entire parasite. For all intensity measurements, a background correction was performed. Dissociation of V-ATPase subunits from the DVM was analyzed by generating a fluorescence intensity profile along a transect spanning the DV lumen and the parasite cytoplasm. A fragmented DV was indicated by the presence of two or more autonomous compartments positive for vacuolar markers. Hemozoin was quantified by polarization microscopy as described previously (5). Stainings with MitoTracker Red CMXRos and Lysosensor Blue DND-167 (Thermo Fisher Scientific) were performed according the manufacturer's instructions. The pH sensor was calibrated by incubating *pm2-sep* schizonts for 30 minutes in media of varying pH supplemented with 50 µM of nigericin, followed by quantitative live fluorescence microscopy. For time-lapse microscopy, *c cKO + crt-mNG* parasites were seeded onto concanavalin A-coated microscopy dishes and imaged at 37°C in intervals of 30 minutes on a temperature-controlled FV1000 confocal microscope (Olympus) (6).

Microscopic quantification of Fluo-CQ fluorescence in live parasites was performed as described previously (7). Parasites at schizont stage protected from light were stained with 500 nM Fluo-CQ

at 37°C for 30 minutes in the presence or absence of 61 nM ConA and washed twice in complete culture medium before being adjusted to a hematocrit of ~50%. 1.5 µl of the cell suspension were transferred onto a glass slide, covered with a cover slip (24 x 65 cm) and immediately imaged over a period of 5 to 7 minutes. Parasites were first identified by DIC using minimal illumination to avoid photobleaching of the probe and were then imaged using 460-500 nm excitation and 512-542 nm emission filters, which overlap well with the spectrum of the fluorescent nitrobenzofurazan moiety of Fluo-CQ (excitation/emission maxima at 467/539 nm). To ensure comparability and identical degrees of photobleaching for all samples, we used a consistent exposure time that did not cause oversaturation of the images. The area of the DV was outlined manually and the fluorescence was measured as background-corrected raw integrated density. Non-specific signal was controlled for by imaging unstained samples. The selected Fluo-CQ concentration and incubation time were optimized to achieve conditions which do not saturate the uptake or the overall capacity of the DV for this probe. This was indicated by our observation that intravacuolar Fluo-CQ fluorescence was linearly correlated with external probe concentration and incubation time at this range (*SI Appendix*, Fig. S11).

Cytosolic pH approximation with BCECF-AM

For cytosolic pH measurements, $DMSO_{18h}$ and RAP_{18h}-treated *B cKO* parasites at schizont stage were released from their host cells by saponin lysis (0.05% in complete culture medium) and washed three times with HEPES-buffered saline (120 mM NaCl; 5 mM KCl, 25 mM HEPES; 20 mM glucose, 1 mM $MgCl_2$; pH 7.1). Washed parasites were stained with the acetoxymethyl ester of BCECF (5 µM; Invitrogen) for 20 minutes at 37°C and washed five times for 5 minutes each in HEPES-buffered saline. Then, parasites were resuspended in 200 µl HEPES-buffered saline and 10 µl of this suspension were added to 490 µl of complete medium adjusted to varying pH values containing either 50 µM nigericin or vehicle only. Following a 20-minute incubation at room temperature, parasite suspensions were transferred to black flat-bottom 96 well plates at a volume of 150 µl per well. Throughout the entire staining procedure, parasites were protected from light to avoid photobleaching of BCECF. Using an EnVision multilabel plate reader (PerkinElmer), samples were excited at wavelengths of 440 and 490 nm and fluorescence was detected at 535 nm. Unstained parasites in complete culture medium served as a background control. The ratio of both background-corrected fluorescence values (490/440) was calculated and cytosolic pH values were inferred from the calibration curves obtained from nigericin-treated parasites.

Quantification of intracellular [^{3}H]CQ accumulation

Briefly, schizont-infected RBCs were magnet-purified and resuspended in 1 ml of prewarmed reaction buffer A (bicarbonate-free RPMI 1640 supplemented with 11 mM glucose, 25 mM HEPES and 2 mM glutamine; pH 7.3) containing 40 nM [^{3}H]CQ (20 Ci/mmol; American Radiolabeled Chemicals) in combination with 61 nM ConA or vehicle at a hematocrit of 25,000 to 30,000 cells per µl, as determined with a Z1-Coulter Particle Counter (Beckman Coulter). Cells were incubated at 37°C and 75 µl aliquots were removed in duplicate at the indicated time points and diluted with an equal volume of ice-cold reaction buffer A. For efflux experiments, infected RBCs were incubated for 15 minutes in reaction buffer A containing 20 nM [^{3}H]CQ. After preloading, cells were washed twice in ice-cold medium and resuspended in prewarmed reaction buffer A containing no or 61 nM ConA. The reaction was held at 37°C and duplicate 150 µl aliquots were removed from the reaction at various time points.

Aliquots from the CQ response assays were spun through a 5:4 mixture of dibutyl phthalate and dioctyl phthalate, and the aqueous phase containing unincorporated [^{3}H]CQ was removed and transferred to a scintillation vial. The cell pellets were recovered by cutting the reaction tubes through the oil layer with a sharp scalpel while squeezing the tubes with tweezers. The tips of the tubes containing the cell pellets were placed in fresh reaction tubes and incubated with 66 µl ethanol and 33 µl NCS tissue solubilizer (Amersham Biosciences) overnight at 55°C. Lysates were then acidified and bleached by the addition of 25 µl 1 N HCl and 25 µl 30% H_2O_2 and transferred to a scintillation vial. Radioactivity of the aqueous phases and the cell lysates was measured using

a Tri-carb 2100TR liquid scintillation counter (Packard). The intracellular drug concentration was calculated from the amount of radio-labeled drug taken up by the cells and by assuming a volume of 75 fl for an infected RBC (8). Drug accumulation was expressed as the ratio of intracellular versus extracellular drug concentration.

Immunoblotting

Parasites were released from their host cells by lysis with 0.03% saponin in phosphate-buffered saline (PBS) at the end of the cycle. Following vigorous washing with PBS, parasites were lysed in 2x Lämmli buffer. Extracts were subjected to sodium dodecyl sulfate polyacrylamide gel electrophoresis and the separated proteins were transferred onto nitrocellulose membranes. Blots were probed with rat anti-mCh (1:5,000; ChromoTek), mouse anti-mNG (1:1,000; ChromoTek), and rat anti-*Pf*BiP (1:1,000) (9) primary antibodies followed by chemiluminescence or fluorescence detection with horseradish peroxidase (1:5,000; Jackson ImmunoResearch)- or fluorophore-coupled secondary antibodies (1:10,000 – 1:20,000; LI-COR), respectively. In place of a previously developed cell fractionation assay based on UV-Vis spectroscopy (10), we performed western blot analysis of saponin-released parasites using rabbit anti-human hemoglobin α (1:1,000; Abcam) primary antibodies to analyze intraparasitic hemoglobin accumulation.

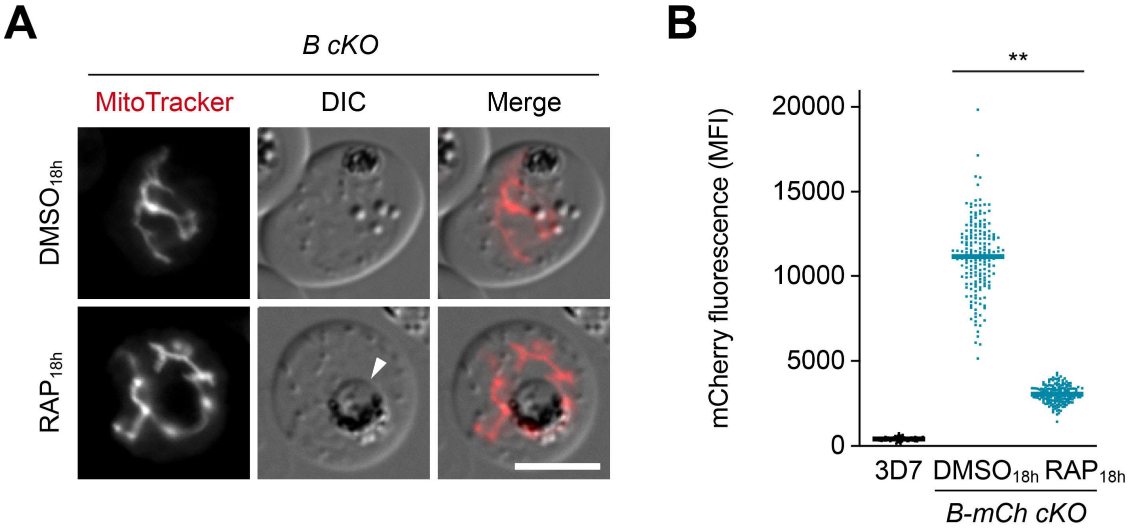

Fig. S1. Delayed rapamycin induction balances loss of function with parasite viability in conditional subunit B knockout parasites. (*A* and *B*) Parasites were tightly synchronized, treated with DMSO or RAP from 18 hours post invasion onward and imaged at schizont stage. (*A*) Delayed RAP induction allows phenotypic analysis of viable parasites. *B cKO* parasites were stained with MitoTracker Red CMXRos. White arrowhead, swollen DV. Note the initiation of segmentation in the RAP_{18h}-treated parasite. Scale bar, 5 µm. (*B*) Microscopic quantification of mCh fluorescence as mean fluorescence intensity (MFI) in *B-mCh cKO* parasites. B11 parasites were included to control for autofluorescence. Individual and mean values; **, $P<0.01$; paired *t*-test; n=180 parasites from 3 independent experiments.

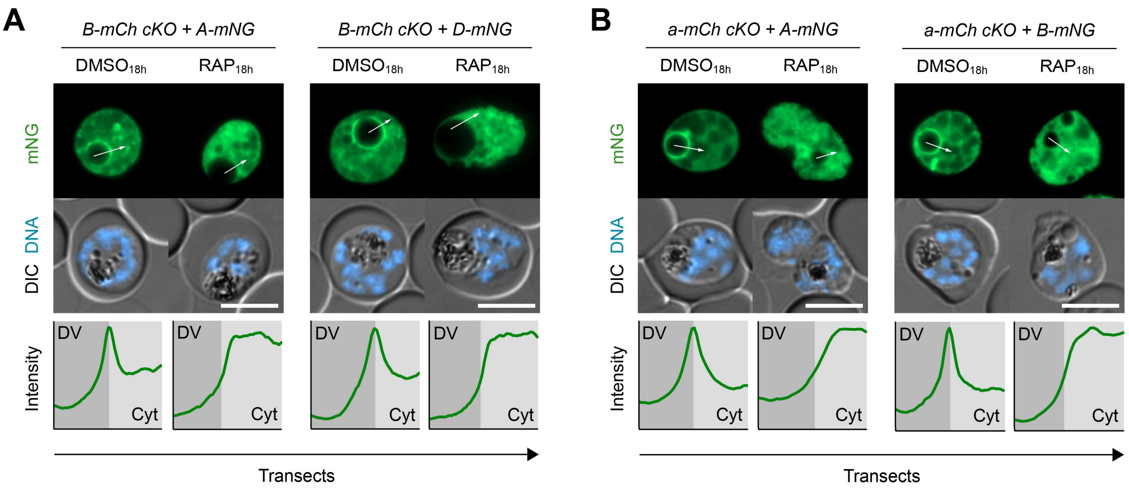

Fig. S2. V_1 disassembly upon loss of subunits B and a**.** (*A* and *B*) *B-mCh cKO* (*A*) and *a-mCh cKO* parasites (*B*) expressing mNG-tagged V_1 subunits were treated with DMSO or RAP from 18 hours post invasion onward and imaged live at schizont stage. Transects (white arrows) spanning the DV lumen and parasite cytoplasm (Cyt) were used to generate intensity profiles of the mNG signal. DNA, Hoechst 33342. Scale bars, 5 µm.

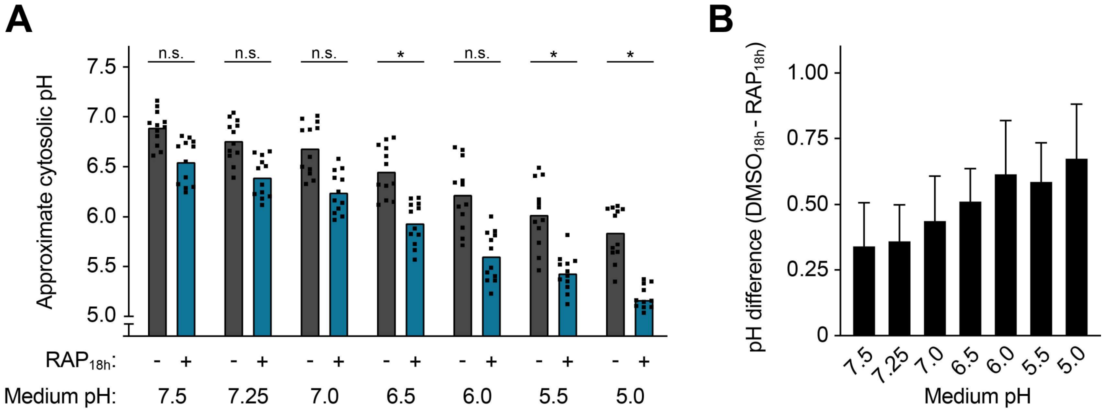

Fig. S3. Loss of subunit B affects cytosolic pH homeostasis. (*A* and *B*) *B cKO* parasites were treated with DMSO or RAP from 18 hours post invasion onward and released from their host cells by saponin treatment at schizont stage. Parasites were stained with the ratiometric pH indicator BCECF acetoxymethyl ester, which was detected on a multilabel plate reader using dual excitation at 440 and 490 nm and emission at 535 nm. The ratio of both fluorescence values (440/490) serves as a readout for intracellular pH and was calibrated with the protonophore nigericin and media of varying pH. (*A*) Cytosolic pH estimates of saponin-released *B cKO* parasites in response to extracellular acidification. Individual and mean values (bars). (*B*) Difference between cytosolic pH estimates of the $DMSO_{18h}$ and RAP_{18h}-treated parasites depicted in *A*. Mean values +/- SEM (bars). *, $P<0.05$; paired *t*-test; n=4 independent experiments with 3 technical replicates each.

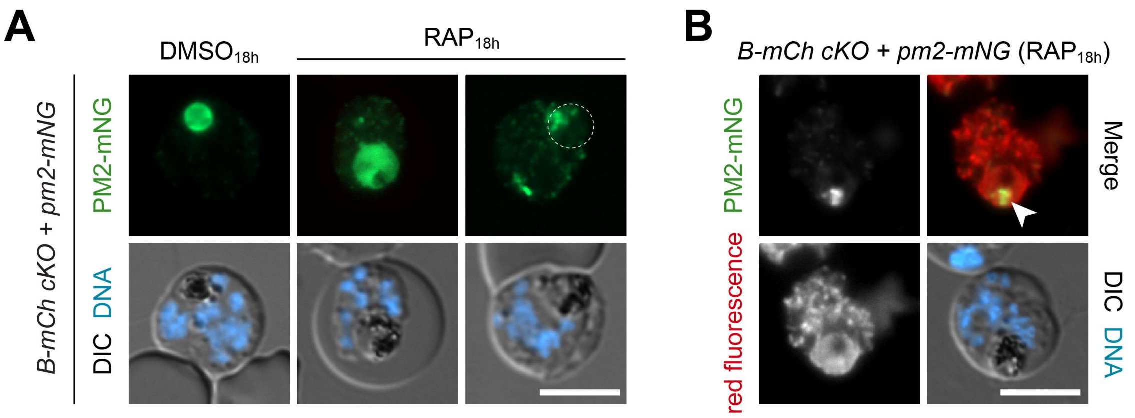

Fig. S4. A DV matrix protein concentrates in vacuolar subregions upon loss of subunit B. *B-mCh cKO* parasites expressing PM2-mNG were synchronized, treated with DMSO or RAP from 18 hours post invasion onward and imaged live at schizont stage. (*A*) Localization of PM2-mNG. In most $DMSO_{18h}$ and RAP_{18h}-treated parasites, PM2-mNG is observed throughout the DV. Occasionally, the signal accumulates in subregions of the swollen DV in RAP_{18h}-treated parasites. Dashed circle, outline of the DV. DNA, Hoechst 33342. Scale bar, 5 µm. (*B*) Co-localization of PM2-mNG with the red signal derived from residual subunit B-mCh protein and endogenous DV fluorescence in RAP_{18h}-treated parasites. Arrowhead, accumulation of PM2-mNG in a DV subregion. DNA, Hoechst 33342. Scale bar, 5 µm.

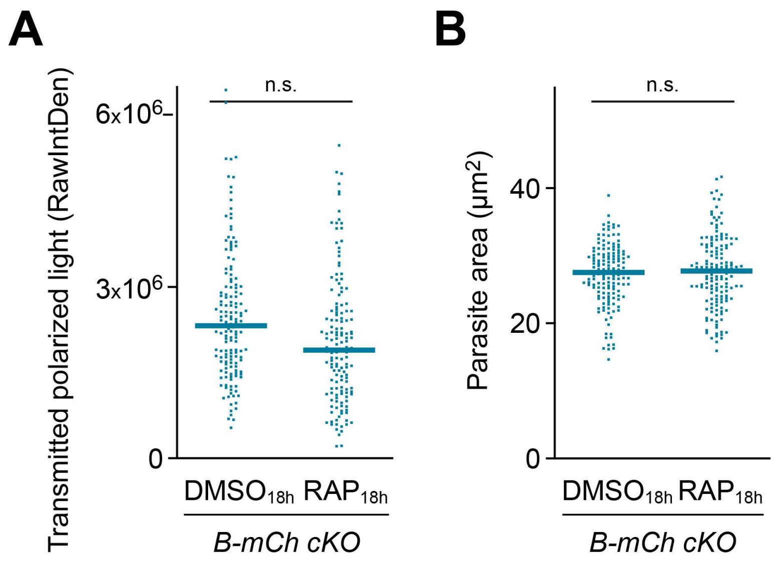

Fig. S5. Subunit B-deficient parasites show a trend towards reduced hemozoin formation. (*A* and *B*) *B-mCh cKO* parasites were treated with DMSO or RAP from 18 hours post invasion onward. At schizont stage, parasites were fixed with methanol and stained with Giemsa. Parasites were imaged by conventional light microscopy and with two crossed polarizers. (*A*) Hemozoin quantification by polarization microscopy. Polarized light transmitted by hemozoin crystals was quantified as raw integrated density (RawIntDen) per parasite. (*B*) Size of the parasites analyzed in *A*, measured as occupied area. Individual and mean values (bars); n.s., non-significant; paired *t*-test; n=150 parasites from 3 independent experiments.

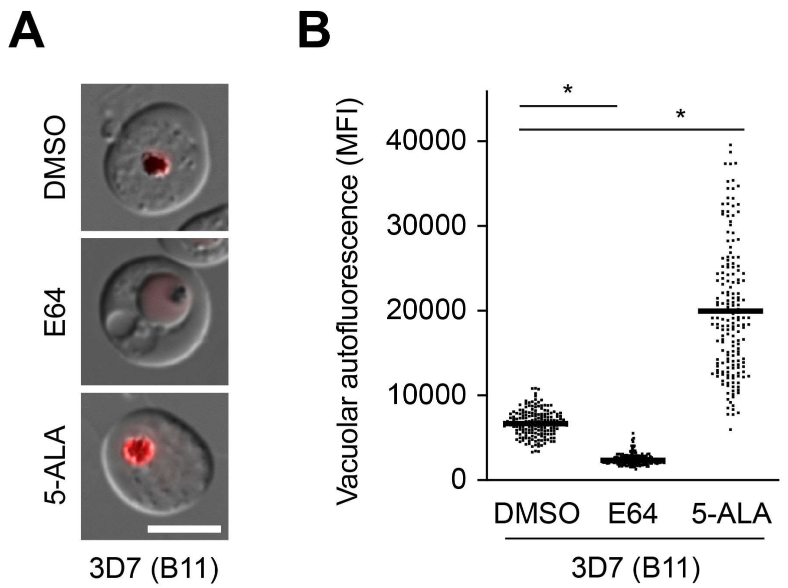

Fig. S6. Autofluorescence as a proxy for heme-derived porphyrins in the DV. (*A* and *B*) B11 parasites were treated with DMSO, 21.7 µM E64 or 10 µM 5-aminolevulinic acid (5-ALA) at 24 hours post invasion and imaged live at schizont stage. DV autofluorescence was recorded with a Texas Red filter cube configuration. (*A*) Representative microscopy images shown as a merge of DIC and red autofluorescence. Scale bar, 5 µm. (*B*) Quantification of red autofluorescence in the DV as mean fluorescence intensity (MFI). *, $P<0.05$; one-way ANOVA and Tukey's multiple comparisons test; n=180 parasites from 3 independent experiments.

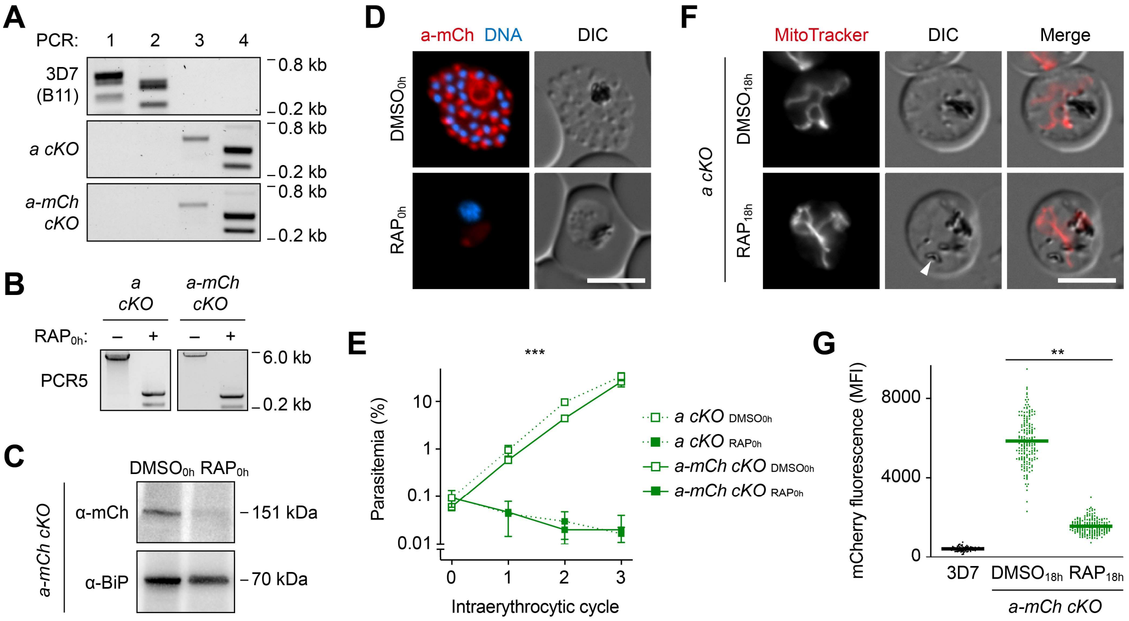

Fig. S7. Subunit a is essential for asexual blood stage development. (*A*) Validation of conditional subunit a knockout parasites. Diagnostic PCRs were performed on genomic DNA from the parental B11 strain as well as from non-tagged (*a cKO*) and mCh-tagged conditional knockout mutants (*a-mCh cKO*). Genetic strategies and primers are as depicted in Fig. 1*B*. (*B*) RAP-induced excision of the subunit a coding sequence. *a cKO* and *a-mCh cKO* cultures were synchronized and treated with DMSO or RAP from the ring stage onward. Genomic DNA was harvested at the end of the cycle and subjected to diagnostic PCR5, as depicted in Fig. 1*B*. (*C* and *D*) RAP-induced loss of mCh-tagged subunit a. *a-mCh cKO* parasites were treated as in *B*. (*C*) Western blot analysis of parasite extracts using primary antibodies against mCh and *Pf*BiP. (*D*) Fluorescence microscopy of live *a-mCh cKO* parasites. DNA, Hoechst 33342. Scale bar, 5 µm. (*E*) Loss of parasite replication in the absence of subunit a. Shown are growth curves of *a cKO* and *a-mCh cKO* parasites treated as in *B*. Mean values +/- SD; ***, P<0.001; Two-way ANOVA; n=3 independent experiments. (*F* and *G*) Delayed RAP induction balances loss of function with parasite viability in conditional subunit a knockout parasites. Parasites were synchronized, treated with DMSO or RAP from 18 hours post invasion onward and imaged at schizont stage. (*F*) Delayed RAP induction allows phenotypic analysis of viable parasites. *a cKO* parasites were stained with MitoTracker Red CMXRos. White arrowhead, scattered hemozoin. Scale bar, 5 µm. (*G*) Microscopic quantification of mCh fluorescence as mean fluorescence intensity (MFI) in *a-mCh cKO* parasites. B11 parasites were included to control for autofluorescence and are identical to those included in *SI Appendix*, Fig. S1*B*. Individual and mean values; **, P<0.01; paired *t*-test; n=180 parasites from 3 independent experiments.

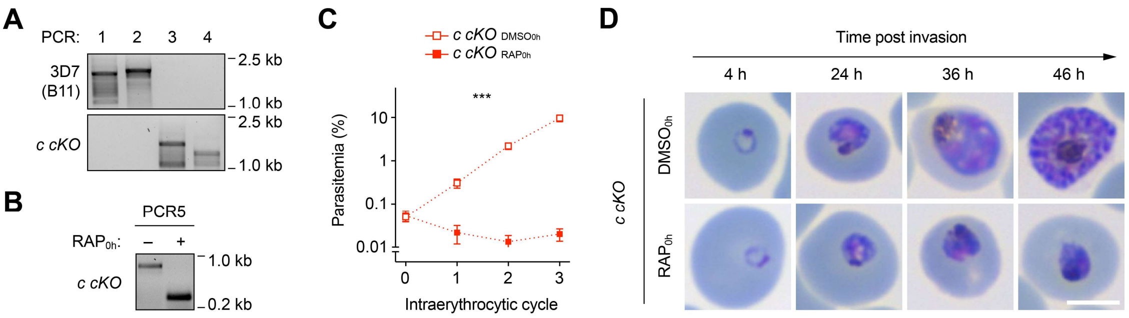

Fig. S8. Subunit c is essential for asexual blood stage development. (*A*) Validation of conditional subunit c knockout parasites. Primers indicated in Fig. 1*B* were used in diagnostic PCRs to analyze genomic DNA from the parental B11 strain as well as from non-tagged *c cKO* parasites. (*B*) RAP-induced excision of the subunit c coding sequence. *c cKO* cultures were synchronized and treated with DMSO or RAP from the ring stage onward. Genomic DNA was harvested at the end of the cycle and subjected to diagnostic PCR5, as depicted in Fig. 1*B*. (*C*) Loss of parasite replication in the absence of subunit c. Shown are growth curves of *c cKO* parasites treated as in *B*. Mean values +/- SD; ***, P<0.001; Two-way ANOVA; n=6 independent experiments. (*D*) Subunit c-deficient parasites arrest development at trophozoite stage. *c cKO* parasites were treated as in *B* and visualized by Giemsa staining throughout the course of one intraerythrocytic cycle. Scale bar, 5 µm.

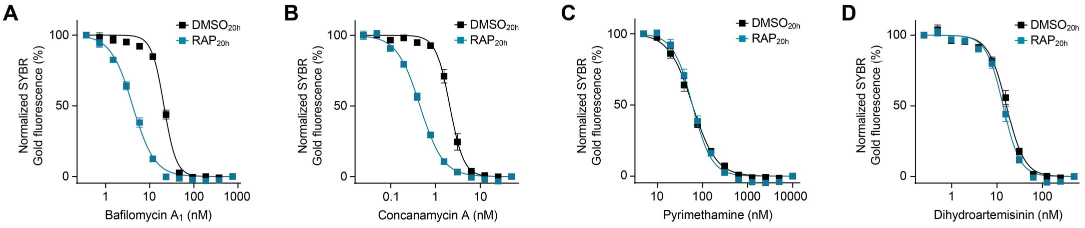

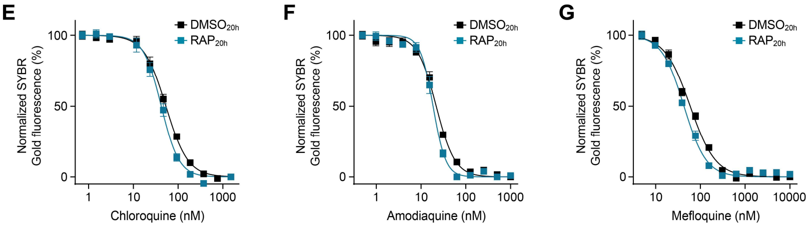

H

B cKO IC_{50} (nM)

Compound	$DMSO_{20h}$	RAP_{20h}	Quotient
Bafilomycin A1	21.0	4.22	4.98 (*)
Concanamycin A	2.19	0.45	4.87 (*)
Pyrimethamine	57.9	62.5	0.93 (n.s.)
Dihydroartemisinin	16.5	13.9	1.19 (n.s.)
Chloroquine	54.3	43.4	1.25 (n.s.)
Amodiaquine	22.1	18.5	1.19 (n.s.)
Mefloquine	59.9	43.1	1.39 (n.s.)

Fig. S9. Susceptibility of subunit B-deficient parasites towards various antimalarials remains unaltered when drugs are added at young schizont stage. (*A* to *G*) Dose-response curves of *B cKO* parasites in the presence of varying concentrations of (*A*) bafilomycin A_1, (*B*) concanamycin A, (*C*) pyrimethamine, (*D*) dihydroartemisinin, (*E*) chloroquine, (*F*) amodiaquine, or (*G*) mefloquine. Synchronized parasites were treated with DMSO or RAP from 20 hours post invasion onward and SYBR Gold fluorescence was recorded 72 hours post invasion. Values were normalized to the lowest non-inhibitory drug concentrations. Non-infected red blood cell cultures were used for background correction. Note that, as opposed to the dose-response experiments shown in Fig. 5, antimalarial compounds were added 36 hours post invasion to account for the rapid action of some of the drugs. Mean values +/- SEM; n=4 independent experiments with 3 technical replicates each. (*H*) IC_{50} values inferred from the dose-response curves in *A* to *G*. The fold-change in sensitivity is expressed as the quotient of IC_{50} values from $DMSO_{20h}$ and RAP_{20h}-treated parasites. n.s., non-significant; *, P<0.05; paired *t*-test; n=4 independent experiments.

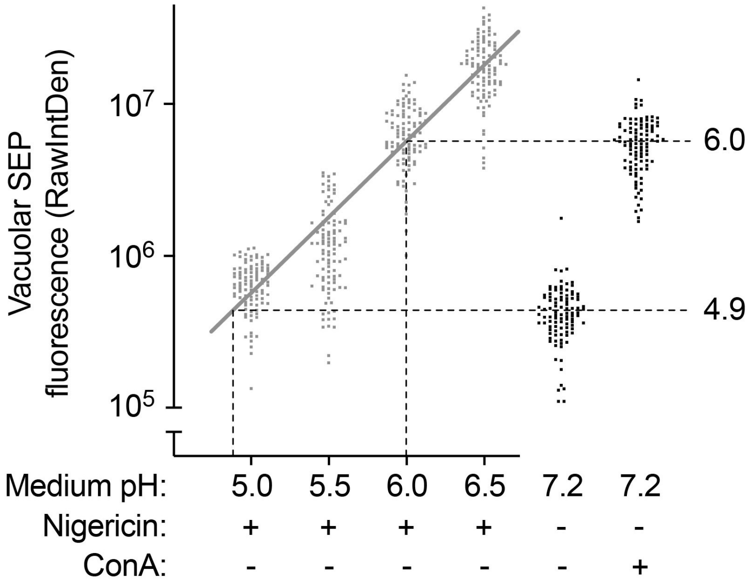

Fig. S10. Calibration of the PM2-SEP sensor and estimation of vacuolar pH. *pm2-sep* parasites at schizont stage were incubated in media of varying pH containing the protonophore nigericin, which causes equilibration of the medium pH with the pH of the DV matrix (gray data sets). Vacuolar SEP fluorescence was then quantified microscopically as background-corrected raw integrated density (RawIntDen). Note that nigericin treatment at pH values lower than 5.0 caused parasite disintegration. Vacuolar pH estimates of DMSO or concanamycin A (ConA)-treated parasites (without nigericin, black data sets) were interpolated from the calibration curve which was generated by non-linear regression using an exponential equation (solid gray line). Shown are individual values. n=99 parasites from 3 independent experiments.

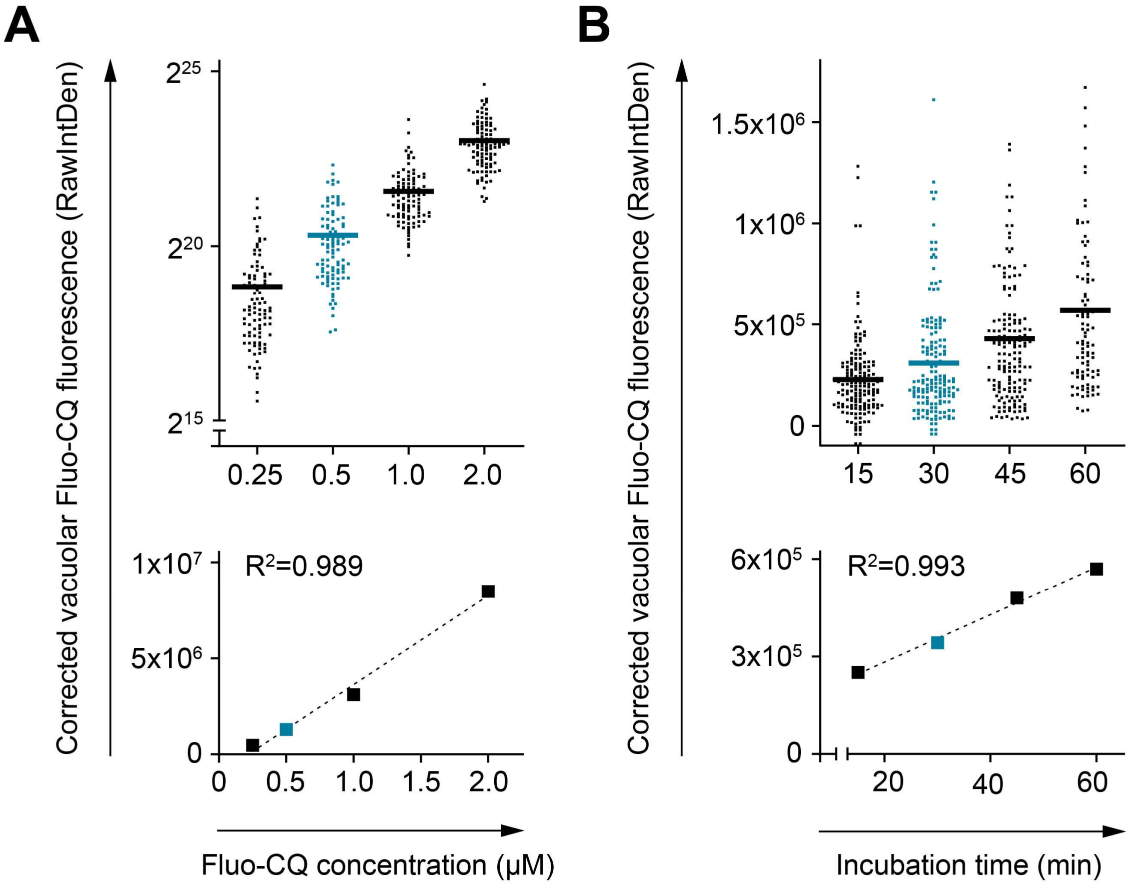

Fig. S11. Influence of external Fluo-CQ concentration and incubation time on intravacuolar fluorescence. (*A* and *B*) B11 parasites at schizont stage were stained with varying concentrations of Fluo-CQ for 30 minutes (*A*) or with 500 nM Fluo-CQ for 15 to 60 minutes (*B*). Fluo-CQ fluorescence in the DV was quantified microscopically as raw integrated density (RawIntDen) and corrected for the mean intensity of unstained controls. Individual and mean values (bars, top). Regression analysis (bottom) indicates that external Fluo-CQ concentration and incubation time are linearly correlated with intravacuolar probe fluorescence. n=99 parasites from 3 independent experiments. Blue color denotes the staining conditions selected for quantitative Fluo-CQ analysis of V-ATPase-deficient parasites.

Table S1. Oligonucleotides used in this study

Oligo name	Purpose[1]	Oligo sequence[2]
**Diagnostic PCR**		
Subunit a 5' F1	PCR1 (581), PCR3 (571)	TTTAAAGTAACAGTACAATGCTCG
Subunit a 5' F2	PCR5 (3719, 584)	AAAGATGGGTATATTTCGATCGG
Subunit a 5' R1	PCR1 (581)	TCCTTTCCATATCATCAATACGC
Subunit a 5' R2	PCR3 (571)	AACTTCGTATAATGTATGCTATACG
Subunit a 3' F1	PCR2 (496)	TTTTACAAGGGAGATGGTATTCCC
Subunit a 3' F2	PCR4 (460)	TAACTTCGTATAGCATACATTATACG
Subunit a 3' R1	PCR2 (496), PCR4 (460)	TTTTTGCATGCAGGTTTTTACCC
Subunit a 3' R2	PCR5 (3719, 584)	CCCCTTCAAAAAAAGGCTAGC
Subunit B 5' F1	PCR1 (693), PCR3 (658)	TATATCTATGGTAATACAAAAAAAGAAAGG
Subunit B 5' F2	PCR5 (2257, 792)	GAGTAAAGAAGTAGTAAATACAAAAGC
Subunit B 5' R1	PCR1 (693)	ATAAACACTTATTACTGTTAACGTTCC
Subunit B 5' R2	PCR3 (658)	AACTTCGTATAATGTATGCTATACG
Subunit B 3' F1	PCR2 (1081)	TACACATCCAATACCTGATTTAACC
Subunit B 3' F2	PCR4 (663)	TAACTTCGTATAGCATACATTATACG
Subunit B 3' R1	PCR2 (1081), PCR4 (663)	GACCACATTATTTCTCTACAACACC
Subunit B 3' R2	PCR5 (2257, 792)	CCACATTATTTCTCTACAACACCC
Subunit c 5' F1	PCR1 (1831), PCR3 (1522)	TAAAGAAGATAGGGAAAAGAAAAAGG
Subunit c 5' F2	PCR5 (750, 233)	TGTGATCCTAATTCAGCTTTTTTTG
Subunit c 5' R1	PCR1 (1831)	GCTGGTGTCACTATAAAATGGG
Subunit c 5' R2	PCR3 (1522)	CTTCTGCTGGTGTCATTTTACC
Subunit c 3' F1	PCR2 (1967)	GTGTAAATGTTGAATTTTTTTTCTTCCC
Subunit c 3' F2	PCR4 (1299)	GTAAAATGACACCAGCAGAAGG
Subunit c 3' R1	PCR2 (1967), PCR4 (1299)	CATAATTGTAGAAACTTGGCTAGG
Subunit c 3' R2	PCR5 (750, 233)	CATTTTGTCTTTCTTCCTTTATATGC
**Cloning guides**		
Subunit a guide F	tag, cKO	attgATTTCAAAATAAATTTTACA
Subunit a guide R	tag, cKO	aaacTGTAAAATTTATTTTGAAAT
Subunit A guide F	tag	attgAAACTTGATGGAAAAATGAT
Subunit A guide R	tag	aaacATCATTTTTCCATCAAGTTT
Subunit B guide1 F	cKO	attgTTGCTGTGTTAGGTGTGCAG
Subunit B guide1 R	cKO	aaacCTGCACACCTAACACAGCAA
Subunit B guide2 F	tag	attgCCACGTCACCATGCCAACTA
Subunit B guide2 R	tag	aaacTAGTTGGCATGGTGACGTGG
Subunit c guide F	cKO	attgATTTGGAACTTTATAGATAG
Subunit c guide R	cKO	aaacCTATCTATAAAGTTCCAAAT
Subunit C guide F	tag	attgTTTAAAGGACACCGAAACGT
Subunit C guide R	tag	aaacACGTTTCGGTGTCCTTTAAA
Subunit D guide F	tag	attgAAAGAAATCTGACAAATTAA
Subunit D guide R	tag	aaacTTAATTTGTCAGATTTCTTT
CRT guide F	tag	attgTGTAATAATTGAATCGACGT
CRT guide R	tag	aaacACGTCGATTCAATTATTACA
PM2 guide F	tag	attgAACGAAAATTTGGAAACCAA
PM2 guide R	tag	aaacTTGGTTTCCAAATTTTCGTT
**Cloning repair templates**		
mCh-linker-HpaI F	cKO (750)	aaatttgttaacggagcaggtgcaggtATGAAGGTGAGCAAGGGCG
mCh-HpaI R	cKO (750)	tttttagtttaacCTTGTACAGCTCGTCCATG
mNG-linker-EcoRV F	tag (747)	aaatttgatatcggagcaggtgcaggtATGGTAAGTAAGGGAGAAGAAGAC
mNG-EcoRV R	tag (747)	tttttagatatcCTTATACAACTCATCCATTCCCATTAC
SEP-linker-EcoRV F	tag (756)	attaatgatatcggagcaggtgcaggtATGGGAAGTAAAGGAGAAGAACTTTTC
SEP-EcoRV R	tag (756)	atatttgatatcTTTGTATAGTTCATCCATGCCATGTG

[1] PCRs 1-5 indicate the relative primer positions as shown in Fig. 1*B*. Oligonucleotides used in the generation of transfection vectors for conditional knockouts or taggings are denoted with cKO or tag, respectively. Expected amplicon sizes (in bp) for wild-type and non-tagged mutants are shown in brackets.

[2] Annealing bases are shown in upper case letters. Overhangs are denoted with lower case letters.

Table S2. Transgenic parasite strains used in this study

Mutant	Parental strain[1]	Description[2]
B11	3D7	Constitutive expression of DiCre recombinase
a cKO	B11	cKO of non-tagged subunit a
a-mCh cKO	B11	cKO of mCh-tagged subunit a
a-mCh cKO + A-mNG	*a-mCh cKO*	mNG-tagging of subunit A in cKO line of mCh-tagged subunit a
a-mCh cKO + B-mNG	*a-mCh cKO*	mNG-tagging of subunit B in cKO line of mCh-tagged subunit a
a-mCh cKO + C-mNG	*a-mCh cKO*	mNG-tagging of subunit C in cKO line of mCh-tagged subunit a
a-mCh cKO + crt-mNG	*a-mCh cKO*	mNG-tagging of CRT in cKO line of mCh-tagged subunit a
a-mCh cKO + pm2-mNG	*a-mCh cKO*	mNG-tagging of PM2 in cKO line of mCh-tagged subunit a
B cKO	B11	Conditional knockout of non-tagged V-ATPase subunit B
B-mCh cKO	B11	Conditional knockout of mCh-tagged V-ATPase subunit B
B-mCh cKO + a-mNG	*B-mCh cKO*	mNG-tagging of subunit a in cKO line of mCh-tagged subunit B
B-mCh cKO + A-mNG	*B-mCh cKO*	mNG-tagging of subunit A in cKO line of mCh-tagged subunit B
B-mCh cKO + C-mNG	*B-mCh cKO*	mNG-tagging of subunit C in cKO line of mCh-tagged subunit B
B-mCh cKO + D-mNG	*B-mCh cKO*	mNG-tagging of subunit D in cKO line of mCh-tagged subunit B
B-mCh cKO + crt-mNG	*B-mCh cKO*	mNG-tagging of CRT in cKO line of mCh-tagged subunit B
B-mCh cKO + pm2-mNG	*B-mCh cKO*	mNG-tagging of PM2 in cKO line of mCh-tagged subunit B
B-mCh cKO + pm2-SEP	*B-mCh cKO*	SEP-tagging of PM2 in cKO line of mCh-tagged subunit B
B-mCh cKO + vp1-mNG	*B-mCh cKO*	mNG-tagging of VP1 in cKO line of mCh-tagged subunit B
c cKO	B11	cKO of non-tagged subunit c
c cKO + a-mNG	*c cKO*	mNG-tagging of subunit a in cKO line of non-tagged subunit c
c cKO + B-mNG	*c cKO*	mNG-tagging of subunit B in cKO line of non-tagged subunit c
c cKO + crt-mNG	*c cKO*	mNG-tagging of CRT in cKO line of non-tagged subunit c
pm2-sep	B11	SEP-tagging of PM2

[1] Recipient strains used as genetic background for parasite transfection.
[2] cKO, conditional knockout; mCh, mCherry; mNG, mNeonGreen; SEP, superecliptic pHluorin.

Sequence S1. Repair template V_0 subunit a-mNG

ACAACCTGACGATGTATATGATCAATATGACGAATACGATGATGATGATGATGATGATAATTATGACGAAAATGAATAT
TTAAATAAAAAAAAAAAAAAAAAGAACAGATGATGATATAGAAGCTCATTTATTAAGTTCTACATATGAAGAGAAAGGATT
ACAAAGTTCATCAGCAGCCATGAGAGGAGGAGGAGCAGGAGAAGAGAATCATCATGAAGAAAACATTTCAGAAATAT
GGATTGAACAATTAATAGAAACAATTGAATTTATATTAGGATTAATTAGTAACACAGCATCATATTTAAGATTATGGGCA
TTATCATTAGCTCATCAACAATTATCATTTGTCTTTTTTGAGCAAACCATTTTAAATTCCTTAAAGAGAAATAGTTTTATG
TCTGTATTAATTAACTTAATATTATTTTCTCAATTATTTTCAATACTTACCATAGCTGTTATATTATGTATGGACACACTG
GAATGTTTCTTACATTCATTAAGATTGCAGTGGGTTGAATTCCAGAACAAGTTTTATAAAGGAGATGGTATTCCCTTCA
AGCCTTTCAACATAAAGAAGTTGTTGAACGAAAATGAGGATATCGGAGCAGGTGCAGGTATGGTAAGTAAGGGAGAA
GAAGACAACATGGCATCATTGCCAGCCACCCACGAGTTGCATATTTTCGGTTCTATTAACGGAGTGGACTTTGATATG
GTTGGTCAGGGTACAGGAAATCCTAATGATGGTTATGAGGAGCTTAATTTGAAATCTACTAAAGGTGACTTGCAGTTT
TCACCTTGGATACTTGTTCCACATATAGGTTACGGTTTTCACCAGTACCTTCCATATCCAGATGGAATGTCTCCATTCC
AGGCTGCTATGGTGGATGGAAGTGGTTATCAAGTACATAGGACCATGCAGTTTGAGGACGGTGCATCTTTAACCGTT
AACTATAGATATACCTATGAAGGATCTCACATTAAAGGTGAAGCCCAAGTGAAAGGTACCGGATTTCCTGCTGACGGT
CCAGTGATGACTAACAGTTTAACTGCCGCAGACTGGTGCCGTTCTAAAAAGACATATCCAAACGATAAGACTATAATT
TCTACCTTTAAATGGTCATACACAACAGGAAATGGAAAACGTTATAGATCAACTGCAAGGACTACTTATACATTCGCTA
AACCAATGGCCGCCAATTATCTTAAAAATCAACCTATGTATGTTTTCCGTAAAACTGAATTAAAGCACTCAAAGACAGA
ACTTAATTTTAAGGAATGGCAAAAAGCATTTACTGACGTAATGGGAATGGATGAGTTGTATAAGGATATCTAACAAGAA
CAAAATCATAAAAGTGTACCATCAACTGTGTAAGTCGTTATAAGCACACACATAAATAAAAATAAAAATGAACATATAAA
TGAATCAATATATATAAATATATATATATATATATATATATATATATATATATATATGTATGTATGTATGTTTGTTTGTTTTTAT
TTATGTGTGCTCATTTTTTTTTTTTTTTTTTCAATTTATGTAATTTAGATATTTTCAAGTTAAAATATTTATGTTGTATTCCTTA
CGTTTTATGTTTATTTATATAATTAATTCTTTTATTATATATTATATTATATTTTATTCCTTTTACTTTTACTTTTTCTTTTTTT
TTTTTTTTTTTTTTTTTTTTTTTTGTTAACATTATAAAAAAAATTAAAAACAGCTAGCCTTTTTTTGAAGGGGTAAAAACCTGC
ATGCAAAAAAAAAAAAAAAAAAAAAAAATATGAACATATAAAGTGCATTCTGTTATATATATATATATATATATATATATAA
TTGCATAATCTAGTTGTGTATTTTTCTCATTTGTTAACATTACAATGTTGACCACCTTATATAAAAAACATATAAGCTTTT
TATAAAGAAAAGATAA

Homology regions, linker, mNG

Sequence S2. Repair template V_0 subunit a cKO

AAAAAAAAAAAAAAAGAACACGAGCAAATGTGCCTATAAATAGGATTGCTATGAGATTATATATTTTGTGGGGAATTCAT
TAAATATATATATATAATAAATAATTTATTATATATATATATATATATATAATATATTTATATATATATATATATATATATATTTTTTT
GTTGATTAATTAAAAATTTTAAGAACCCTGAAAATGATAAAGTGTGTTTAATTATATTTGGAAGAAGGGTTAAAAAATTT
TGAATTTTTTTTTTTTTTTTATTTAATCAAAGATATATATCTATATATCTATATATATATATAATACATTTATTTGTTTAAAGA
TGGGTATATTTCGATCGGAGATTATGAAACACGGGACATTAGTGCTGCCTTCTGATAGGGCAAGGGAATATTTGGATT
GCTTAGGAAAAGAAGTGGATATACAATTCATAGATATGTAAATAAAAAAAATAATATACAATAACTTCGTATAGCATACA
TTATACGAAGTTATTATATATGTATATATATATATATTTATATATTTTATATTCTTTTAGGAATGAAAAAACAATGAAGCGT
CAGTATAAAAAATACATTCAAAGAATAGATGATATGGAAAGAATTTTGAGATTCTTGGAGGAGAACATTAATAAGTTGC
CTAATGTAAAGATAAAGAAAAGTAAGATTGACAATTTCCTTGAGCATGATAACATATACGAGTTAGACCAGGTGGAAG
AGAGTTTAAATAGACTTCACGTACAATTTGTGAGATTTTGCAACAATAACAAAGACTTAATTGACGAGAAGAATAACGC
CATAGAGGAGAAACACGTAATATTGACAGCCTTAAACCAACTTTCTCCTGGTTTTATTCGTGGTGTTGGAGGTTTGCG
TGGTTCAGTAATAGGTGGTGATCAGCAACAACAGCACCATCATAACAATAACAATAATAACAACATAGTATCTCCTTTT
GATGAAGGAATTGAGGAGAACAACATATCATTGTCTACACATATAATGAAAGATGGAATAAATATGATGTTCACCAACA
TAAGTGGAGTTATAAAAACTAAAGATCAAGAATCATTCTCACGTACAATTTTTAGGGCATTTCGTGGAAATACCTACAC
CTACTTTCAGAACATTGATGAGGACGCCGATGACAATGATTTACTTAAGAACGTGGAAGAGTTGGAAAGTCTTAAGTC
TGCTGGTGATACCAATAACATGTATAAGAATAACAAACAGCATGATACCTTTTCTGATAAATTGGACGGAAAAGATAAA
AACGATAAAAAAAAAAAGAAAGACTTGAAATCAGTATTTGTGGTGTACTGCCAGGGATCAGCACAGTCAAACATATAT
GACAAGATAATGAAAATATGTAAGGCCTACGATGTAAAAACTTATGATTGGCCTAGGACCTACGAACATGCCAAAAAG
CGTCTTAAAGAATTGCGTGAAATTATTAACGATAAGGAGAAAGCCTTAAAAGCATATGAAGAGTACTTCATAAACGAGA
TATTTGTTTTGATAAATGTTGTGGAACCTAACAAGAATTCACTTATAGAGGAATGGAAATTATTTTGTAAAAAAGAAAGG
CACATTTATAATAATTTAAACTACTTCGAAGGAAGTGACATAACATTAAGATGCGATTGTTGGTATTCAGCAAATGACG
AGGAGAAAATTAGACATATACTTATAAACAAGAGTTCTAACGATCTTGTGTCTGCATTATTGCTTTCTGATAAAATTTTG
AGACCAAACGTAAGTCCACCTACATATATTAAGACCAATGAGTTTACCAAAAGTTATCAGTCTATGGTAGATACTTATG
GTGTGCCAAGGTACGGTGAGATTAATCCAGCCATTTCTACCATAATAACCTTTCCTTTTCTTTTTGGTATTATGTATGG
AGACGTGGGACATGGACTTTGTATTTTCTTATTCGCCTTATTTCTTATTATTATGAACAATAAAGTTAAGAATAAAAAAA
ATAATAATGAAATGGTGACCATGCTTTTCGATGGACGTTACATGTTGTTGTTAATGGGATTCTTCGCAGTTTACGCAGG
ATTCTTATATAATGATTTTTTCAGTATGCCATTAAACCTTTTCAGTTCAATGTTCATGCTTGACAAGCAGGTAGACAATA
TGGAGTACTATAAGAGGAGAGAGATAACCGATTCTGCAACCGGAGAGGTACAATATGCATATCCTTACATATTTGGTT
TTGATTGTAAATGGTTAGGTGCCGAGAATGAGTTGACTTACATTAATTCATTTAAAATGAAGTTCTCAATAATAATAGGT
TTTATACACATGACTTTTGGTGTTTTAATGAAAGGATTTAATGCATTACATTTCAAGCGTAAGATGGACTTCTTTTTTGA
ATTTTTGCCTCAACTTGTAATGATGCTTTCTATGATTGGATACTTGGTTTTCTTGATTATTTATAAGTGGGTAACACCTG
TTGGATACGGAGGTTTTCAGAAACAGGGTATTATAAATACAATTATAAATATGTATTTAATGAAAGAAATTAACTCAACT
AATCAATTCTACCCATACCAGTCTATAATACAGATTCTTTTATTAAGTTTGTTTGTGTTATGCATACCTTTCATGTTCATA
TGTAAACCAGCAATAAGGACTTACCATATAATGAAAGAAAAACAGAAAATTAATGATCATAACGTACATAAGAAGAATA
GAAGAGGTAAAAAGGCCATGGCCAAGAGGAATAACAATAATAATAACAATAACAACAACCAAAACGACAACAAAAATA
TGCTTTTGACAAACCAGAATTTTAATGAGGCCGGTGCAAAAGAGATGATTAATGCCTTCCACCACAATGCCAAAACAG
GTGGAATTTTATTATCTACAAACTCTAAGAAAAAGATTGTGACCTCAGACGGTAATCATAACAATAATCTTTTATCTATG
CATAAACGTAACACTATGAACAACAAAATAAGTTCATCTCCTTATCATAATGAACAACCAGATGATGTTTACGATCAGT
ACGATGAGTACGATGATGATGACGATGATGATAATTACGACGAGAATGAATATCTTAATAAAAAAAAAAAAGAAGAGAA
CCGACGACGACATTGAGGCACACTTATTATCTAGTACATATGAAGAAAAGGGTTTGCAATCTTCATCAGCCGCCATGA
GAGGAGGTGGTGCAGGTGAGGAGAATCACCATGAAGAGAATATTAGTGAGATTTGGATAGAGCAATTAATAGAAACT
ATAGAGTTCATTTTAGGTCTTATATCAAATACCGCTAGTTATTTAAGGTTATGGGCCCTTAGTTTAGCTCACCAGCAGT
TGTCTTTCGTGTTCTTCGAACAGACTATATTAAACTCATTAAAAAGGAACTCATTTATGTCTGTTCTTATAAATTTGATAT
TGTTTAGTCAGCTTTTCTCAATATTAACTATTGCAGTAATATTGTGCATGGATACTCTTGAGTGTTTCCTTCACTCTTTG
CGTTTACAGTGGGTTGAATTTCAGAATAAATTCTATAAAGGAGACGGAATACCTTTTAAGCCATTTAACATTAAAAAGC
TTTTGAATGAGAATGAGGTTAACTAAAATATAACTTCGTATAGCATACATTATACGAAGTTATCAAGAACAAAATCATAA
AAGTGTACCATCAACTGTGTAAGTCGTTATAAGCACACACATAAATAAAAATAAAAATGAACATATAAATGAATCAATAT
ATATAAATATATATATATATATATATATATATATATATATATATATGTATGTATGTATGTTTGTTTGTTTTTATTTATGTGTGCT
CATTTTTTTTTTTTTTTTTCAATTTATGTAATTTAGATATTTTCAAGTTAAAATATTTATGTTGTATTCCTTACGTTTTATGTT
TATTTATATAATTAATTCTTTTATTATATATTATATTATATTTTATTCCTTTTACTTTTACTTTTTCTTTTTTTTTTTTTTTTTTT
TTTTTTTTTTT

Homology regions, recodonized gene, artificial intron, *loxP* sites

Sequence S3. Repair template V_0 subunit a-mCh cKO

AAAAAAAAAAAAAAAGAACACGAGCAAATGTGCCTATAAATAGGATTGCTATGAGATTATATATTTTGTGGGGAATTCAT
TAAATATATATATATAATAAATAATTTATTATATATATATATATATATATAATATATTTATATATATATATATATATATATATTTTTT
GTTGATTAATTAAAAATTTTAAGAACCCTGAAAATGATAAAGTGTGTTTAATTATATTTGGAAGAAGGGTTAAAAAATTT
TGAATTTTTTTTTTTTTTTTATTTAATCAAAGATATATATCTATATATCTATATATATATATAATACATTTATTTGTTTAAAGA
TGGGTATATTTCGATCGGAGATTATGAAACACGGGACATTAGTGCTGCCTTCTGATAGGGCAAGGGAATATTTGGATT
GCTTAGGAAAAGAAGTGGATATACAATTCATAGATATGTAAATAAAAAAAATAATATACAATAACTTCGTATAGCATACA
TTATACGAAGTTATTATATATGTATATATATATATATTTATATATTTTATATTCTTTTAGGAATGAAAAAACAATGAAGCGT
CAGTATAAAAAATACATTCAAAGAATAGATGATATGGAAAGAATTTTGAGATTCTTGGAGGAGAACATTAATAAGTTGC
CTAATGTAAAGATAAAGAAAAGTAAGATTGACAATTTCCTTGAGCATGATAACATATACGAGTTAGACCAGGTGGAAG
AGAGTTTAAATAGACTTCACGTACAATTTGTGAGATTTTGCAACAATAACAAAGACTTAATTGACGAGAAGAATAACGC
CATAGAGGAGAAACACGTAATATTGACAGCCTTAAACCAACTTTCTCCTGGTTTTATTCGTGGTGTTGGAGGTTTGCG
TGGTTCAGTAATAGGTGGTGATCAGCAACAACAGCACCATCATAACAATAACAATAATAACAACATAGTATCTCCTTTT
GATGAAGGAATTGAGGAGAACAACATATCATTGTCTACACATATAATGAAAGATGGAATAAATATGATGTTCACCAACA
TAAGTGGAGTTATAAAAACTAAAGATCAAGAATCATTCTCACGTACAATTTTTAGGGCATTTCGTGGAAATACCTACAC
CTACTTTCAGAACATTGATGAGGACGCCGATGACAATGATTTACTTAAGAACGTGGAAGAGTTGGAAAGTCTTAAGTC
TGCTGGTGATACCAATAACATGTATAAGAATAACAAACAGCATGATACCTTTTCTGATAAATTGGACGGAAAAGATAAA
AACGATAAAAAAAAAAAGAAAGACTTGAAATCAGTATTTGTGGTGTACTGCCAGGGATCAGCACAGTCAAACATATAT
GACAAGATAATGAAAATATGTAAGGCCTACGATGTAAAAACTTATGATTGGCCTAGGACCTACGAACATGCCAAAAAG
CGTCTTAAAGAATTGCGTGAAATTATTAACGATAAGGAGAAAGCCTTAAAAGCATATGAAGAGTACTTCATAAACGAGA
TATTTGTTTTGATAAATGTTGTGGAACCTAACAAGAATTCACTTATAGAGGAATGGAAATTATTTTGTAAAAAAGAAAGG
CACATTTATAATAATTTAAACTACTTCGAAGGAAGTGACATAACATTAAGATGCGATTGTTGGTATTCAGCAAATGACG
AGGAGAAAATTAGACATATACTTATAAACAAGAGTTCTAACGATCTTGTGTCTGCATTATTGCTTTCTGATAAAATTTTG
AGACCAAACGTAAGTCCACCTACATATATTAAGACCAATGAGTTTACCAAAAGTTATCAGTCTATGGTAGATACTTATG
GTGTGCCAAGGTACGGTGAGATTAATCCAGCCATTTCTACCATAATAACCTTTCCTTTTCTTTTTGGTATTATGTATGG
AGACGTGGGACATGGACTTTGTATTTTCTTATTCGCCTTATTTCTTATTATTATGAACAATAAAGTTAAGAATAAAAAAA
ATAATAATGAAATGGTGACCATGCTTTTCGATGGACGTTACATGTTGTTGTTAATGGGATTCTTCGCAGTTTACGCAGG
ATTCTTATATAATGATTTTTTCAGTATGCCATTAAACCTTTTCAGTTCAATGTTCATGCTTGACAAGCAGGTAGACAATA
TGGAGTACTATAAGAGGAGAGAGATAACCGATTCTGCAACCGGAGAGGTACAATATGCATATCCTTACATATTTGGTT
TTGATTGTAAATGGTTAGGTGCCGAGAATGAGTTGACTTACATTAATTCATTTAAAATGAAGTTCTCAATAATAATAGGT
TTTATACACATGACTTTTGGTGTTTTAATGAAAGGATTTAATGCATTACATTTCAAGCGTAAGATGGACTTCTTTTTTGA
ATTTTTGCCTCAACTTGTAATGATGCTTTCTATGATTGGATACTTGGTTTTCTTGATTATTTATAAGTGGGTAACACCTG
TTGGATACGGAGGTTTTCAGAAACAGGGTATTATAAATACAATTATAAATATGTATTTAATGAAAGAAATTAACTCAACT
AATCAATTCTACCCATACCAGTCTATAATACAGATTCTTTTATTAAGTTTGTTTGTGTTATGCATACCTTTCATGTTCATA
TGTAAACCAGCAATAAGGACTTACCATATAATGAAAGAAAAACAGAAAATTAATGATCATAACGTACATAAGAAGAATA
GAAGAGGTAAAAAGGCCATGGCCAAGAGGAATAACAATAATAATAACAATAACAACAACCAAAACGACAACAAAAATA
TGCTTTTGACAAACCAGAATTTTAATGAGGCCGGTGCAAAAGAGATGATTAATGCCTTCCACCACAATGCCAAAACAG
GTGGAATTTTATTATCTACAAACTCTAAGAAAAAGATTGTGACCTCAGACGGTAATCATAACAATAATCTTTTATCTATG
CATAAACGTAACACTATGAACAACAAAATAAGTTCATCTCCTTATCATAATGAACAACCAGATGATGTTTACGATCAGT
ACGATGAGTACGATGATGATGACGATGATGATAATTACGACGAGAATGAATATCTTAATAAAAAAAAAAAAGAAGAGAA
CCGACGACGACATTGAGGCACACTTATTATCTAGTACATATGAAGAAAAGGGTTTGCAATCTTCATCAGCCGCCATGA
GAGGAGGTGGTGCAGGTGAGGAGAATCACCATGAAGAGAATATTAGTGAGATTTGGATAGAGCAATTAATAGAAACT
ATAGAGTTCATTTTAGGTCTTATATCAAATACCGCTAGTTATTTAAGGTTATGGGCCCTTAGTTTAGCTCACCAGCAGT
TGTCTTTCGTGTTCTTCGAACAGACTATATTAAACTCATTAAAAAGGAACTCATTTATGTCTGTTCTTATAAATTTGATAT
TGTTTAGTCAGCTTTTCTCAATATTAACTATTGCAGTAATATTGTGCATGGATACTCTTGAGTGTTTCCTTCACTCTTTG
CGTTTACAGTGGGTTGAATTTCAGAATAAATTCTATAAAGGAGACGGAATACCTTTTAAGCCATTTAACATTAAAAAGC
TTTTGAATGAGAATGAGGTTAACGGAGCAGGTGCAGGTATGAAGGTGAGCAAGGGCGAGGAGGATAACATGGCCAT
CATCAAGGAGTTCATGCGCTTCAAGGTGCACATGGAGGGCTCCGTGAACGGCCACGAGTTCGAGATCGAGGGCGAG
GGCGAGGGCCGCCCCTACGAGGGCACCCAGACCGCCAAGCTGAAGGTGACCAAGGGTGGCCCCCTGCCCTTCGC
CTGGGACATCCTGTCCCCTCAGTTCATGTACGGCTCCAAGGCCTACGTGAAGCACCCCGCCGACATCCCCGACTACT
TGAAGCTGTCCTTCCCCGAGGGCTTCAAGTGGGAGCGCGTGATGAACTTCGAGGACGGCGGCGTGGTGACCGTGA
CCCAGGACTCCTCCCTGCAGGACGGCGAGTTCATCTACAAGGTGAAGCTGCGCGGCACCAACTTCCCCTCCGACGG
CCCCGTAATGCAGAAGAAGACCATGGGCTGGGAGGCCTCCTCCGAGCGGATGTACCCCGAGGACGGCGCCCTGAA
GGGCGAGATCAAGCAGAGGCTGAAGCTGAAGGACGGCGGCCACTACGACGCTGAGGTCAAGACCACCTACAAGGC
CAAGAAGCCCGTGCAGCTGCCCGGCGCCTACAACGTCAACATCAAGTTGGACATCACCTCCCACAACGAGGACTAC
ACCATCGTGGAACAGTACGAACGCGCCGAGGGCCGCCACTCCACCGGCGGCATGGACGAGCTGTACAAGGTTAAC
TAAAATATAACTTCGTATAGCATACATTATACGAAGTTATCAAGAACAAAATCATAAAAGTGTACCATCAACTGTGTAAG
TCGTTATAAGCACACACATAAATAAAAATAAAAATGAACATATAAATGAATCAATATATATAAATATATATATATATATAT
ATATATATATATATATATATGTATGTATGTATGTTTGTTTGTTTTTATTTATGTGTGCTCATTTTTTTTTTTTTTTTCAATTT
ATGTAATTTAGATATTTTCAAGTTAAAATATTTATGTTGTATTCCTTACGTTTTATGTTTATTTATATAATTAATTCTTTTAT
TATATATTATATTATATTTTATTCCTTTTACTTTTACTTTTTCTTTTTTTTTTTTTTTTTTTTTTTTTTTTTT

Homology regions, recodonized gene, artificial intron, *loxP* sites, linker, mCh

Sequence S4. Repair template V_0 subunit c cKO

AAGTTAAACCTGTTTTTATTTTATTTATTTATTTTTATATTATTTCCACCATTTTTTTTTTAAATATATTATATATATATATATAA
ATAAATAAAAAAATATATATAAATAATTTTTAATTTTAAAATAAAATAATATAAATAAATAAAAAAATAAATATATGTATATAT
TATAAACACATATACATATAATATATATATATATATATATATATAATATATATATATATATATATATATAATATATATATATGTAATTA
TAAGTTAACAATTTCATTTTATTTTTATTTTACTATTCTTTTATTTAATTTTTTTTTTTTTTTTTTTTTTTTTGTGCCGAAAATGC
GACAATGTGATCCTAATTCAGCTTTTTTTGGATTTATGGGAATTGCAGCTTCCTCAATATTTTCTAGTAAATAAAAAAAA
TAATATACAATAACTTCGTATAGCATACATTATACGAAGTTATTATATATGTATATATATATATATTTATATATTTTATATT
CTTTTAGATTTGGGTGCAGCTTTCGGAACTGCAAAATCTGGTGTAGGAGTGTGCTCAGTGGGAGTTATGAGACCAGA
CCTTATAATGAAGAGTATTCTTCCAGTTGTGATGGCAGGAGTTCTTGGAATATACGGAATTATTATGTCAATACTTATAT
ATGGTAAAATGACACCAGCAGAAGGATATTCAACTTTTGCTGGATATGCACATCTTTCATCAGGACTTATAGTTGGACT
TTCATCTTTGGCCGCCGGTTTAGCTATTGGTATAGTTGGAGATGCAGGTGTGCGTGCTAACGCCCAACAGAACAGAT
TATTTATTGGAATGATACTTATTTTAGTTTTTTCTGAAACCCTTGCATTATACGGACTTATTATTGGAATATATATTAGTA
TTGCCGAAACACCAAAGTTGTGTACTCCATATAACGTTGATATCTAAAATATAACTTCGTATAGCATACATTATACGAA
GTTATTTTTTTTTTTTTTTATTAACAAAATTAAATATATATAAATAAATATATATATATATATATGCAGACAAAATAGACAA
ACTTTATGATATGCATATAAAGGAAGAAAGACAAAATGGTATTTTGTTTTCCCCATTCATATCCTATTTTTGTTTTTTTTT
TTTTTCTTTCTTTTTTTTTTTTTAGTTTATGAGAATATTAAAAAAAAAAAAAAAAAAAAAATACATAAAGATGAATAAATTTACA
AATGTATATATTTAATATATATTTGTTGAAACCATATGATAATAGAATGGGATGAAGAAAAAGAGAAAAAATCTTTATTTA
TATAAGAATTATGATAATTATAATAATAATAACCGAAGAAAAAAAAAAAAAAAAAAAAAAGAAGAAAAAGAAAACAACAT

Homology regions, recodonized gene, artificial intron, *loxP* sites

Sequence S5. Repair template V_1 subunit A-mNG

TAGTGATATTTTACAACAAGAAAGTGACTTGAATGATATTGTTCAACTAGTAGGAAAGGATTCATTATCAGAAGACCAA
AAAGTTGTTATGGAAGTAGCCAAAATTATTAGAGAAGATTTTCTTCAACAAAATGCATTTAGCGATTATGATTATATGTG
CCCATTACAAAAAACAGTTGGTATGATGAGAATTATTTGCCACTTTTATGCTCAATGCTTAAGAACATTACAAGAATAT
GACTCAAGAGAAAGAAAAATTGGTTGGGGATCTATATATAATACATTAAGACCAACTATAAATAAAATTACACATATGA
AATTTGAAAACCCAAAAAATTCAGATGAATATTTCAAAAAGTATTTTAAGGCACTTGAAGAAGAAATAACAGTAGGTTTA
AGAAATCTAATGGAGAAGGTTATCGGAGCAGGTGCAGGTATGGTAAGTAAGGGAGAAGAAGACAACATGGCATCATT
GCCAGCCACCCACGAGTTGCATATTTTCGGTTCTATTAACGGAGTGGACTTTGATATGGTTGGTCAGGGTACAGGAA
ATCCTAATGATGGTTATGAGGAGCTTAATTTGAAATCTACTAAAGGTGACTTGCAGTTTTCACCTTGGATACTTGTTCC
ACATATAGGTTACGGTTTTCACCAGTACCTTCCATATCCAGATGGAATGTCTCCATTCCAGGCTGCTATGGTGGATGG
AAGTGGTTATCAAGTACATAGGACCATGCAGTTTGAGGACGGTGCATCTTTAACCGTTAACTATAGATATACCTATGA
AGGATCTCACATTAAAGGTGAAGCCCAAGTGAAAGGTACCGGATTTCCTGCTGACGGTCCAGTGATGACTAACAGTT
TAACTGCCGCAGACTGGTGCCGTTCTAAAAAGACATATCCAAACGATAAGACTATAATTTCTACCTTTAAATGGTCATA
CACAACAGGAAATGGAAAACGTTATAGATCAACTGCAAGGACTACTTATACATTCGCTAAACCAATGGCCGCCAATTA
TCTTAAAAATCAACCTATGTATGTTTTCCGTAAAACTGAATTAAAGCACTCAAAGACAGAACTTAATTTTAAGGAATGGC
AAAAAGCATTTACTGACGTAATGGGAATGGATGAGTTGTATAAGGATAACTAATAGGATCAAAAAAAAAAAAAAAAAAGA
AAAAAAAAAAAAAAATGTGATTTATTTATTAGTTGAATAATTAAAGCTATTAATACTTAATTGTATATGATATATGTCATATA
ACAAAATGAAATTTATATAATATATATATATATATATATATATATATATTAATACTTAATGAAGGTTTTAAAAAAAAATTATAAT
ATTTATATTGGCACAAATATGAATAAGAAAAGAAAAAATAATATTATGATCTTTTTTTTTTTTTTACAATACATTTTTAAAAT
ATATTAATATATATACATGTAAGTATAATAATTTGATATAATCGATGTTAAAAGATAACATAATTTTTTTTTTTATAGCAATAT
TTTTAATATTATTTTTTATATTACATTTTTAATTATTATATATATATATATTAATAAA

Homology regions, linker, mNG

Sequence S6. Repair template V_1 subunit B-mNG

CGGCAAGAGAAGAAGTACCAGGTAGAAGAGGATATCCAGGATATATGTATAGTGATTTATCCACCATATATGAAAGAG
CTGGAAGAGTTGAAGGACGTAATGGTAGTATAACACAATTTCCTATATTAACAATGCCTAATGATGATATTACACATCC
AATACCTGATTTAACCGGATATATTACAGAAGGTCAAATATTTGTAGACAGAAATTTATATAATAGACAAATATATCCTC
CAATCAATGTCTTACCATCTTTATCGCGTTTAATGAAAAGTGGTATTGGACATAATATGACCAGAATCGATCATCCATA
TGTATCTGATCAATTATATAGTAATTATGCTATTGCTCAAGATGTAAAAGCAATGAAAGCAGTAATTGGTGAAGAAGCA
CTTTCAAATGATGATATTCTATATCTTGAATTCTTAGATAAATTCGAAAAGAGATTTATTACACAAAATACATATGAATGT
AGAGATATTTATCAATCATTGGATATCGCATGGGAACTTTTAAGAATCTTTCCAGAAGATATGCTTAAAAAAATTAAAAC
AGACATTTTATCCAAATATTATCCTCGACATCACGCAAACGTTATCGGAGCAGGTGCAGGTATGGTAAGTAAGGGAGA
AGAAGACAACATGGCATCATTGCCAGCCACCCACGAGTTGCATATTTTCGGTTCTATTAACGGAGTGGACTTTGATAT
GGTTGGTCAGGGTACAGGAAATCCTAATGATGGTTATGAGGAGCTTAATTTGAAATCTACTAAAGGTGACTTGCAGTT
TTCACCTTGGATACTTGTTCCACATATAGGTTACGGTTTTCACCAGTACCTTCCATATCCAGATGGAATGTCTCCATTC
CAGGCTGCTATGGTGGATGGAAGTGGTTATCAAGTACATAGGACCATGCAGTTTGAGGACGGTGCATCTTTAACCGT
TAACTATAGATATACCTATGAAGGATCTCACATTAAAGGTGAAGCCCAAGTGAAAGGTACCGGATTTCCTGCTGACGG
TCCAGTGATGACTAACAGTTTAACTGCCGCAGACTGGTGCCGTTCTAAAAAGACATATCCAAACGATAAGACTATAAT
TTCTACCTTTAAATGGTCATACACAACAGGAAATGGAAAACGTTATAGATCAACTGCAAGGACTACTTATACATTCGCT
AAACCAATGGCCGCCAATTATCTTAAAAATCAACCTATGTATGTTTTCCGTAAAACTGAATTAAAGCACTCAAAGACAG
AACTTAATTTTAAGGAATGGCAAAAAGCATTTACTGACGTAATGGGAATGGATGAGTTGTATAAGGATAACTAAGGAAA
ATCAAAAAAACATATATGAAATGATAAATTGTGCCTAAAAAAAAAAAAAAAAAAAATACATATATATATATATATATATA
TATAATATATATGTTTATACATATATGTAATAATTTATTTTTTTTCAAGGTGTTAAAAAAATGTACCCCCACTTAAATTTTT
TTTTTTTGAATAATCTTATCTATTTAATTCATTTTTAAATAAAAAAAAAAAAAAGAAACAACCTTTTCATTATATATGTTT
GTATATATCTATATGTATTGTTTTATTTTATTCATTTAATAATAATAGTATATCCGTTACATCATTTTTTTTTTTTTTTTTTT
TTTTATATATATATTCCAAAATTTTATTGTTCTTATATATATATAATATATATATATATATATATATATTAAACAAATAAGTAGCTT
ATTATATATCATATTATATAATATTATGTAGAATTTTTTTTTTTTTTTTTTCTTAATTTTGTTTAATAATTTAAAATTTTCTAA
TAACTAAAAAATAAGTCAAATAAAATCAAAAAAAAATAAAATAATAAATGCATAAAAAATTAAACAATAAATGTACATATA
TATTTGTATATTT

Homology regions, linker, mNG

Sequence S7. Repair template V_1 subunit B cKO

AAAGCTTGAATAATAATGTAGATTAAAAACATTATATATATATATACAAATATCCAAAAAAATTATATACACATATATAAA
CATATATTTATATATATGTATATATATTTTTCAGTACATTTATTATTATCTTAAAATTTCAGTTATAATATTATAAATGTTCT
GGATATAAATAATAATTTGATATAAAATATATAAAAAGAAGAAAAAAAAAAAAAAATAATAATAATCCATCTTTTAGTCAT
TTTGTATTTAAAAATAAAAAAAAGATTATAAATAATAAAATATATAATGAGTAAAGAAGTAGTAAATACAAAAGCTGAAG
CATCTCGTGTTAATGCTTTAGCAGCTGTGAGGAATTATAAAGTGTGTCCACGACTAGAATATAAAACCATTTCAGGTAA
ATAAAAAAAAATAATATACAATAACTTCGTATAGCATACATTATACGAAGTTATTATATATGTATATATATATATATTTATAT
ATTTTATATTCTTTTAGGTGTTCAAGGTCCATTAGTTATTATAGAGGATGTTAAGTTCCCAAAGTACTCAGAAATTGTGA
CAATTCATCTTTCAGATAACACCACCAGGCAGGGTCAAATACTTGAGGTGTGTGGTAAGAAGGCCGTGATTCAGGTG
TTTGAAGGAACCTCAGGAATTGACAACAAGAACAGTTACGTAGAGGTAAGTGGAGATATTTTAAAGATGCCAATGAGT
GATGAGATGTTAGGACGTGTATTCAACGGTTCAGGTAAACCAATTGATAAAGGACCAAACATTCTTGCAGACGATTAC
TTGGACATAAACGGTAATCCTATAAACCCACAGTGCAGGGTATATCCTAAAGAAATGATTCAGACTGGTATTAGTACC
ATAGATGTAATGAACTCTATTGTTAGAGGACAAAAAATTCCACTTTTCTCAGCCGCCGGTCTTCCTCATAATGAGATTG
GAGCCCAGATTTGCAGGCAAGCATCTTTGGTTCAAGGAAAGGACGTACTTGATCATTCAGATGATAACTTTGCAGTAG
TATTTGGTGCAATGGGTGTGAACATGGAGACTGCTAGATACTTTCGTCAAGATTTTGAAGAGAATGGAAAGATGGAAA
GAGTATGCCTTTTTTTGAACCTTGCTAATGATCCTACCATTGAGAGAATACTTACTCCACGTATAGCTTTAACTACTGC
CGAGTATTTAGCATTTGAGAAAGAAATGCATGTTTTCGTGATTTTGACTGACATGTCTTCATACGCTGATGCCTTAAGA
GAGGTGAGTTCTGCTAGGGAGGAGGTGCCAGGTCGTCGTGGTTATCCAGGTTATATGTATTCAGACTTGTCTACTATT
TACGAGAGAGCAGGAAGAGTGGAAGGACGTAATGGTTCTATAACCCAATTCCCAATTCTTACCATGCCAAATGACGAT
ATAACCCATCCAATACCAGATCTTACTGGATACATTACAGAAGGACAGATTTTCGTGGATAGGAATTTGTATAACAGAC
AAATTTACCCTCCTATTAACGTGTTACCTTCTCTTTCAAGACTTATGAAGTCAGGAATAGGTCATAACATGACAAGGAT
TGACCACCCTTATGTATCAGATCAATTGTATTCAAATTATGCCATAGCACAAGATGTGAAAGCCATGAAGGCTGTGATA
GGTGAAGAGGCCTTGTCTAATGATGACATATTATACCTTGAATTTCTTGATAAGTTTGAAAAAAGGTTCATAACCCAAA
ATACATACGAATGTAGGGATATTTACCAGTCACTTGACATTGCCTGGGAGCTTTTAAGGATATTCCCTGAGGATATGTT
AAAAAAGATAAAGACAGACATTTTGTCAAAATACTATCCTAGACACCATGCCAATGTTAACTAAAATATAACTTCGTATA
GCATACATTATACGAAGTTATGGAAAATCAAAAAAACATATATGAAATGATAAATTGTGCCTAAAAAAAAAAAAAAAAAA
ATACATATATATATATATATATATATATATATAATATATATGTTTATACATATATGTAATAATTTATTTTTTTTCAAGGTGTTAA
AAAAATGTACCCCCACTTAAATTTTTTTTTTTTTGAATAATCTTATCTATTTAATTCATTTTTAAATAAAAAAAAAAAAAAG
AAACAACCTTTTCATTATATATGTTTGTATATATCTATATGTATTGTTTTATTTTATTCATTTAATAATAATAGTATATCCG
TTACATCATTTTTTTTTTTTTTTTTTTTTTTTTTATATATATTCCAAAATTTTATTGTTCTTATATATATAATATATATATATATA
TATATATATT

Homology regions, recodonized gene, artificial intron, *loxP* sites

Sequence S8. Repair template V_1 subunit B-mCh cKO

AAAGCTTGAATAATAATGTAGATTAAAAACATTATATATATATATACAAATATCCAAAAAAATTATATACACATATATAAA
CATATATTTATATATATGTATATATATTTTTCAGTACATTTATTATTATCTTAAAATTTCAGTTATAATATTATAAATGTTCT
GGATATAAATAATAATTTGATATAAAATATATAAAAAGAAGAAAAAAAAAAAAAAAATAATAATAATCCATCTTTTAGTCAT
TTTGTATTTAAAAATAAAAAAAAGATTATAAATAATAAAATATATAATGAGTAAAGAAGTAGTAAATACAAAAGCTGAAG
CATCTCGTGTTAATGCTTTAGCAGCTGTGAGGAATTATAAAGTGTGTCCACGACTAGAATATAAAACCATTTCAGGTAA
ATAAAAAAAAATAATATACAATAACTTCGTATAGCATACATTATACGAAGTTATTATATATGTATATATATATATATTTATAT
ATTTTATATTCTTTTAGGTGTTCAAGGTCCATTAGTTATTATAGAGGATGTTAAGTTCCCAAAGTACTCAGAAATTGTGA
CAATTCATCTTTCAGATAACACCACCAGGCAGGGTCAAATACTTGAGGTGTGTGGTAAGAAGGCCGTGATTCAGGTG
TTTGAAGGAACCTCAGGAATTGACAACAAGAACAGTTACGTAGAGGTAAGTGGAGATATTTTAAAGATGCCAATGAGT
GATGAGATGTTAGGACGTGTATTCAACGGTTCAGGTAAACCAATTGATAAAGGACCAAACATTCTTGCAGACGATTAC
TTGGACATAAACGGTAATCCTATAAACCCACAGTGCAGGGTATATCCTAAAGAAATGATTCAGACTGGTATTAGTACC
ATAGATGTAATGAACTCTATTGTTAGAGGACAAAAAATTCCACTTTTCTCAGCCGCCGGTCTTCCTCATAATGAGATTG
GAGCCCAGATTTGCAGGCAAGCATCTTTGGTTCAAGGAAAGGACGTACTTGATCATTCAGATGATAACTTTGCAGTAG
TATTTGGTGCAATGGGTGTGAACATGGAGACTGCTAGATACTTTCGTCAAGATTTTGAAGAGAATGGAAAGATGGAAA
GAGTATGCCTTTTTTTGAACCTTGCTAATGATCCTACCATTGAGAGAATACTTACTCCACGTATAGCTTTAACTACTGC
CGAGTATTTAGCATTTGAGAAAGAAATGCATGTTTTCGTGATTTTGACTGACATGTCTTCATACGCTGATGCCTTAAGA
GAGGTGAGTTCTGCTAGGGAGGAGGTGCCAGGTCGTCGTGGTTATCCAGGTTATATGTATTCAGACTTGTCTACTATT
TACGAGAGAGCAGGAAGAGTGGAAGGACGTAATGGTTCTATAACCCAATTCCCAATTCTTACCATGCCAAATGACGAT
ATAACCCATCCAATACCAGATCTTACTGGATACATTACAGAAGGACAGATTTTCGTGGATAGGAATTTGTATAACAGAC
AAATTTACCCTCCTATTAACGTGTTACCTTCTCTTTCAAGACTTATGAAGTCAGGAATAGGTCATAACATGACAAGGAT
TGACCACCCTTATGTATCAGATCAATTGTATTCAAATTATGCCATAGCACAAGATGTGAAAGCCATGAAGGCTGTGATA
GGTGAAGAGGCCTTGTCTAATGATGACATATTATACCTTGAATTTCTTGATAAGTTTGAAAAAAGGTTCATAACCCAAA
ATACATACGAATGTAGGGATATTTACCAGTCACTTGACATTGCCTGGGAGCTTTTAAGGATATTCCCTGAGGATATGTT
AAAAAAGATAAAGACAGACATTTTGTCAAAATACTATCCTAGACACCATGCCAATGTTAACGGAGCAGGTGCAGGTAT
GAAGGTGAGCAAGGGCGAGGAGGATAACATGGCCATCATCAAGGAGTTCATGCGCTTCAAGGTGCACATGGAGGGC
TCCGTGAACGGCCACGAGTTCGAGATCGAGGGCGAGGGCGAGGGCCGCCCCTACGAGGGCACCCAGACCGCCAA
GCTGAAGGTGACCAAGGGTGGCCCCCTGCCCTTCGCCTGGGACATCCTGTCCCCTCAGTTCATGTACGGCTCCAAG
GCCTACGTGAAGCACCCCGCCGACATCCCCGACTACTTGAAGCTGTCCTTCCCCGAGGGCTTCAAGTGGGAGCGCG
TGATGAACTTCGAGGACGGCGGCGTGGTGACCGTGACCCAGGACTCCTCCCTGCAGGACGGCGAGTTCATCTACAA
GGTGAAGCTGCGCGGCACCAACTTCCCCTCCGACGGCCCCGTAATGCAGAAGAAGACCATGGGCTGGGAGGCCTC
CTCCGAGCGGATGTACCCCGAGGACGGCGCCCTGAAGGGCGAGATCAAGCAGAGGCTGAAGCTGAAGGACGGCG
GCCACTACGACGCTGAGGTCAAGACCACCTACAAGGCCAAGAAGCCCGTGCAGCTGCCCGGCGCCTACAACGTCAA
CATCAAGTTGGACATCACCTCCCACAACGAGGACTACACCATCGTGGAACAGTACGAACGCGCCGAGGGCCGCCAC
TCCACCGGCGGCATGGACGAGCTGTACAAGGTTAACTAAAATATAACTTCGTATAGCATACATTATACGAAGTTATGG
AAAATCAAAAAAACATATATGAAATGATAAATTGTGCCTAAAAAAAAAAAAAAAAAAAATACATATATATATATATATATAT
ATATATAATATATATGTTTATACATATATGTAATAATTTATTTTTTTTCAAGGTGTTAAAAAAATGTACCCCCACTTAAATT
TTTTTTTTTTTGAATAATCTTATCTATTTAATTCATTTTTAAATAAAAAAAAAAAAAAGAAACAACCTTTTCATTATATATGT
TTGTATATATCTATATGTATTGTTTTATTTTATTCATTTAATAATAATAGTATATCCGTTACATCATTTTTTTTTTTTTTTTT
TTTTTTATATATATATTCCAAAATTTTATTGTTCTTATATATATATAATATATATATATATATATATATATATT

Homology regions, recodonized gene, artificial intron, *loxP* sites, linker, mCh

Sequence S9. Repair template V_1 subunit C-mNG

TATAGAAACAGAATATCTAACAACACTTATAGCTTATGTACCTAAAAATTCTATAGATGATTGGTTAAATAATTATGAAA
AATTTTCATCCTATGTTGTACCTAGATCTACAGAACAATTTAAAGATTTAATAGATAAAGATGGAAATACATTATGGAAA
GTTTTTGTTTTTAAGAAATTTGCAGAAGATTTTAAAAAAGAAGCAAAAGTTAAAAAATTTGTTGTAAAATCATTTAAATAT
GATGAAAAACAATATAATGATATGATGGAATCGAGAACAAAAGTAGAAGCAGAAATCATAAGACAAGAAACTTTTCTAA
GACGCATGTGCTTAGCCGCTTTTTCAGATATATTTATTGCATTCATTCATATTAATATACTTCGAGTCTTTTGTGAATCT
GTATTACGATTTGGTGTTCCACCTAATTTTGCTTCATTTAGTATAAGAATTAATGGAGAAAGTAAAGAAAAAAAAGTCA
GAAAAAAATTATACGACATTTTTTCATCATCTGATTCTATAGGAAAGAATTATATAAAAAGATCTGATGAAAATGATGAA
GAAATATATCCATATGTATCAGTATCATTTAAGATAGATATCGGAGCAGGTGCAGGTATGGTAAGTAAGGGAGAAGAA
GACAACATGGCATCATTGCCAGCCACCCACGAGTTGCATATTTTCGGTTCTATTAACGGAGTGGACTTTGATATGGTT
GGTCAGGGTACAGGAAATCCTAATGATGGTTATGAGGAGCTTAATTTGAAATCTACTAAAGGTGACTTGCAGTTTTCA
CCTTGGATACTTGTTCCACATATAGGTTACGGTTTTCACCAGTACCTTCCATATCCAGATGGAATGTCTCCATTCCAGG
CTGCTATGGTGGATGGAAGTGGTTATCAAGTACATAGGACCATGCAGTTTGAGGACGGTGCATCTTTAACCGTTAACT
ATAGATATACCTATGAAGGATCTCACATTAAAGGTGAAGCCCAAGTGAAAGGTACCGGATTTCCTGCTGACGGTCCA
GTGATGACTAACAGTTTAACTGCCGCAGACTGGTGCCGTTCTAAAAAGACATATCCAAACGATAAGACTATAATTTCTA
CCTTTAAATGGTCATACACAACAGGAAATGGAAAACGTTATAGATCAACTGCAAGGACTACTTATACATTCGCTAAACC
AATGGCCGCCAATTATCTTAAAAATCAACCTATGTATGTTTTCCGTAAAACTGAATTAAAGCACTCAAAGACAGAACTT
AATTTTAAGGAATGGCAAAAAGCATTTACTGACGTAATGGGAATGGATGAGTTGTATAAGGATATCTGAAATTGGAAA
GGCTATAAATTGGGTATACCAATATGTATAAATGTATATAAATGTATATAAATGTATATATGCATATATATATATATATAT
ATATATATATATATGTGTATGTTATTTTTTATGTGTTAATGAACCAATTTATTATTATTACAACCAAATCCAGGAATATCA
ACCCATGATATGTGCTACACATGTTTAAAAGTATTAGATACCAATATGAAGGTGAAGAAGAAAAGAATTATTTTTGTAA
GAATAATATTTTTTTACGCAACTATTATATTTTACATGTAACTATTACTTTATTTTATTTTTTCCATTTATTAAAAAAAAAAG
TAGCACAAATTAGAAGAAGATATTATTTTATTTGTACCCATGATTGTTACAGTACTCTACTTTCTAATATATATAAGCTAAAA
ATATAGTAAAAATAATTAATTGTTAACATTAATTTTATATGAGAACTTATAATAGAAAAATATAATTATATATATATATATA
TATATATATATATTTATTAATTTTATATATATTTTAATTGTTATCTTTTTTCTTTTTTTTTTTTTTTTTTTTTTTTTCCGCTTTA
TTAATAAGATGAACT

Homology regions, linker, mNG

Sequence S10. Repair template V_1 subunit D-mNG

AGAGTTAACGCACTAAATAATGTAAGACCAACAAAAAATAAAATAAATATGTGAAGAATAACTTTCATAGTTTTTTATAA
TTAACTAAATTAATATAAACATTTGATATGATATTTTTCTTATTATTTTGTATTAATATAGATTGTGCTCCCAAGGTTAGAT
GGAGGTATAAATTATATTATAAAAGAATTAGACGAAATAGAAAGAGAAGAATTCTACAGGTAAATATAACAGTGATATT
ATTATACAAACGTGTAATATATATATATATATATATATATATATATATATATATGGATGTATGTATATATGTATGTATTTTTTTTTT
TTCTTTCTTTTTTCAAATTTAAAACTCATTCTTATTATTTGTCACATTCACCTTTTAGGTTAAAAAAAATAAAGGAAAAAAA
GTCAGACAAGTTAAAAGATTCTAATATAGACACTGATGCAGACGGAGATTACAACGCCTCAAAAAGACAATATAATTAC
GCTTGTACCCAAAAAGACGACGATATTATATTTGATATCGGAGCAGGTGCAGGTATGGTAAGTAAGGGAGAAGAAGA
CAACATGGCATCATTGCCAGCCACCCACGAGTTGCATATTTTCGGTTCTATTAACGGAGTGGACTTTGATATGGTTGG
TCAGGGTACAGGAAATCCTAATGATGGTTATGAGGAGCTTAATTTGAAATCTACTAAAGGTGACTTGCAGTTTTCACCT
TGGATACTTGTTCCACATATAGGTTACGGTTTTCACCAGTACCTTCCATATCCAGATGGAATGTCTCCATTCCAGGCTG
CTATGGTGGATGGAAGTGGTTATCAAGTACATAGGACCATGCAGTTTGAGGACGGTGCATCTTTAACCGTTAACTATA
GATATACCTATGAAGGATCTCACATTAAAGGTGAAGCCCAAGTGAAAGGTACCGGATTTCCTGCTGACGGTCCAGTG
ATGACTAACAGTTTAACTGCCGCAGACTGGTGCCGTTCTAAAAAGACATATCCAAACGATAAGACTATAATTTCTACCT
TTAAATGGTCATACACAACAGGAAATGGAAAACGTTATAGATCAACTGCAAGGACTACTTATACATTCGCTAAACCAAT
GGCCGCCAATTATCTTAAAAATCAACCTATGTATGTTTTCCGTAAAACTGAATTAAAGCACTCAAAGACAGAACTTAAT
TTTAAGGAATGGCAAAAAGCATTTACTGACGTAATGGGAATGGATGAGTTGTATAAGGATATCTAAAATTTTTAATTTAT
CATTTGATATATTTATTATACATGTTCAATTTATTATTATTTTATTTTATATTTCTTTCCTTCCTTTTTTTTTTTTTTTTTTTT
TTTTTTTTTGAATAAATTACAACTATTTAAATACAATTAAAAAAGTAAAAAAAAATTGAATAAAATAAAAAAATATATGTTG
ATTAAATTAAATATATATATATATTAGATATATTTACTCACGTCTTGCACTGAGCAAGCATTATAAATATATAAACAGTATTT
TCTTTATCATTCTATAAGATATTGTGAAAATAACATAAAGTGTAATAATATTCCTTAAAAATAAAGAATCACAAAAAAAAA
AAAAAAAAAAAAAAAGAGATTCTCTTTTATTTTTATATAATTCTACCTTTTTGTAATT

Homology regions, linker, mNG

Sequence S11. Repair template CRT-mNG

TTTTTTTTCACAATATACATTTAAATGTTTATGATGGTACAACGTATCATATTTTATAATAATTTTATGCATTCATGTATAT
TATTTTTACTTTTTAATTTTATAGGGTGATGTTGTAAGAGAACCAAGATTATTAGATTTCGTAACTTTGGTAAGTGTGAA
ATTAAAAAATGAATTTTTTTTTTTTTTTTTTATGAACAAAATAATGTGTATATAATATGTGTGTAATATCAAATGGCTTGTT
CGTTCATAAATATTTATATTTTTTTTTTTTTTTTTTTTTTTTTTTTTTTTTTACAGTTTGGCTACCTATTTGGTTCTATAATTTA
CCGTGTAGGAAATATTATCTTAGAAAGTAATACAAAAATAAGATAAAAAAATATAATATATAAAATATGTATATTGTTCTT
ATATATTTTGTTCATATATATATATATATATATATATATTTTTATATTTCCATCTGTCTTTTTATTCTATTGTTATAATTTATC
ATAAATTTTTTTTTAAATTTGCTTACATTAGGAAAAAAAAATGAGAAATGAAGAAAATGAAGATTCCGAAGGAGAATTAACG
AATGTGGATTCTATTATAACACAAGTTATCGGAGCAGGTGCAGGTATGGTAAGTAAGGGAGAAGAAGACAACATGGC
ATCATTGCCAGCCACCCACGAGTTGCATATTTTCGGTTCTATTAACGGAGTGGACTTTGATATGGTTGGTCAGGGTAC
AGGAAATCCTAATGATGGTTATGAGGAGCTTAATTTGAAATCTACTAAAGGTGACTTGCAGTTTTCACCTTGGATACTT
GTTCCACATATAGGTTACGGTTTTCACCAGTACCTTCCATATCCAGATGGAATGTCTCCATTCCAGGCTGCTATGGTG
GATGGAAGTGGTTATCAAGTACATAGGACCATGCAGTTTGAGGACGGTGCATCTTTAACCGTTAACTATAGATATACC
TATGAAGGATCTCACATTAAAGGTGAAGCCCAAGTGAAAGGTACCGGATTTCCTGCTGACGGTCCAGTGATGACTAA
CAGTTTAACTGCCGCAGACTGGTGCCGTTCTAAAAAGACATATCCAAACGATAAGACTATAATTTCTACCTTTAAATGG
TCATACACAACAGGAAATGGAAAACGTTATAGATCAACTGCAAGGACTACTTATACATTCGCTAAACCAATGGCCGCC
AATTATCTTAAAAATCAACCTATGTATGTTTTCCGTAAAACTGAATTAAAGCACTCAAAGACAGAACTTAATTTTAAGGA
ATGGCAAAAAGCATTTACTGACGTAATGGGAATGGATGAGTTGTATAAGGATAACTAAAATTATATCATTTATTTTTTTA
TATTTTTTATATTTTTTTTATTTTTCATGTTTTTTTCTTTATTTTTTTTTTTTTTTTTTTTCCTTTTTTTTTTTAATAAATCATTTT
CCTAATATTTATATTTGCTATCGCATTACACTTTATAAGGAATAATGTTGAAGGTATAGAGATCTCTTTATTATTATTAAG
ATGTAAATTTTTTATGAAATTTTGTACTTTTTTAGTATTTATATTATATACACATAAATATATATATATATTTATTTATTGATAT
TTGAAAAAAGAATAAGGAAATAAAAAAAGGGAAAAAAAAAAAAGTTAAAAGTATATATATATATATATATATATATATATTTA
TTTATTTATATATATATTATTATGTATGTTTATAATTTATATATATATATGTCATAAAAAGAAATATAATTATATATATATATATATTA
TATTATATATATATATATAAATTTTTTTAAATTATAAACTATTGTTATTGTTAAATTTAATTTTTAATTCATTAAAAATCAGATT
ACCAAAGATTTTTTCCATTCTTCAATCATGATAATAGAAATGAGAAGAAGCAAAAAAAGAAAAAAAAAAAAAAATTC

Homology regions, linker, mNG

Sequence S12. Repair template PM2-mNG

TATCAATAGGTTCAGTAGATCCAATTGTTGTTGAATTAAAAAACCAAAACAAAATTGAAAATGCTCTTTTCACCTTTTAC
TTACCTGTACATGATAAACATACAGGATTCTTAACCATTGGTGGTATTGAAGAAAGATTTTATGAAGGACCACTAACTT
ACGAAAAATTAAACCACGATTTATATTGGCAAATAACTTTAGATGCACACGTTGGAAATATAATGTTAGAAAAAGCAAA
CTGTATTGTAGATAGTGGTACTAGTGCCATTACTGTACCAACTGACTTTTTAAATAAAATGTTGCAGAATTTAGATGTTA
TCAAAGTCCCATTCTTACCTTTCTATGTAACTCTTTGTAACAACAGCAAATTACCAACTTTTGAATTTACCTCAGAAAAT
GGTAAATACACATTAGAACCTGAATACTACCTTCAACACATAGAAGATGTTGGTCCAGGATTATGTATGCTTAATATCA
TAGGATTAGATTTTCCAGTACCAACCTTTATTCTAGGTGACCCATTCATGAGAAAATATTTTACCGTCTTTGATTATGAT
AATCAGAGTGTTGGTATTGCTCTTGCTAAAAAGAATTTAGTTATCGGAGCAGGTGCAGGTATGGTAAGTAAGGGAGAA
GAAGACAACATGGCATCATTGCCAGCCACCCACGAGTTGCATATTTTCGGTTCTATTAACGGAGTGGACTTTGATATG
GTTGGTCAGGGTACAGGAAATCCTAATGATGGTTATGAGGAGCTTAATTTGAAATCTACTAAAGGTGACTTGCAGTTT
TCACCTTGGATACTTGTTCCACATATAGGTTACGGTTTTCACCAGTACCTTCCATATCCAGATGGAATGTCTCCATTCC
AGGCTGCTATGGTGGATGGAAGTGGTTATCAAGTACATAGGACCATGCAGTTTGAGGACGGTGCATCTTTAACCGTT
AACTATAGATATACCTATGAAGGATCTCACATTAAAGGTGAAGCCCAAGTGAAAGGTACCGGATTTCCTGCTGACGGT
CCAGTGATGACTAACAGTTTAACTGCCGCAGACTGGTGCCGTTCTAAAAAGACATATCCAAACGATAAGACTATAATT
TCTACCTTTAAATGGTCATACACAACAGGAAATGGAAAACGTTATAGATCAACTGCAAGGACTACTTATACATTCGCTA
AACCAATGGCCGCCAATTATCTTAAAAATCAACCTATGTATGTTTTCCGTAAAACTGAATTAAAGCACTCAAAGACAGA
ACTTAATTTTAAGGAATGGCAAAAAGCATTTACTGACGTAATGGGAATGGATGAGTTGTATAAGGATAACTAAAAACGA
TTATTAGGATACTAAAGAAAAAACATAATTATATAAGTATAATTATATATATTCACATTTTTTATATATGAATATAAGAAAA
CATATTTTATCCATAATGAACCATTTCATAGAAATTTAAATAATTAAAAAATATTTTTTATTTTTTTTTATTTTTATTTTTATT
TTTTTATTTTATTTTTTTTTTTTTTTTATTTTTTTTTTTACATTCTCCGTATTCTTGTAAAAGAATATAATGATAATAAAAACA
TTTTTTTATTATGATTTTATAATTTTTTATATTTAATTTTTTTTTATTTTTTTTCATAGCATTAATTAATATATATATATATATATA
TTAGTTATATTTGTGAAATAACTTTTCTTTAATAACCTAACACAAGAGTGAGTTCATAAAATTTTATATTTTGCCATATAT
ATATATATATATATATATATATATATATATATATATATATATATATATACATATATATATTTATGTATATATGTGTTTTTCCAATAT
TGGTTCACATTTCTTCTTTTTTCACTCGAAAATATTTGACTTATTATATTTTATATTATATATATATAAAAAAAAAAAAAAAAT
TGTTAAAG

Homology regions, linker, mNG

Sequence S13. Repair template PM2-SEP

TATCAATAGGTTCAGTAGATCCAATTGTTGTTGAATTAAAAAACCAAAACAAAATTGAAAATGCTCTTTTCACCTTTTAC
TTACCTGTACATGATAAACATACAGGATTCTTAACCATTGGTGGTATTGAAGAAAGATTTTATGAAGGACCACTAACTT
ACGAAAAATTAAACCACGATTTATATTGGCAAATAACTTTAGATGCACACGTTGGAAATATAATGTTAGAAAAAGCAAA
CTGTATTGTAGATAGTGGTACTAGTGCCATTACTGTACCAACTGACTTTTTAAATAAAATGTTGCAGAATTTAGATGTTA
TCAAAGTCCCATTCTTACCTTTCTATGTAACTCTTTGTAACAACAGCAAATTACCAACTTTTGAATTTACCTCAGAAAAT
GGTAAATACACATTAGAACCTGAATACTACCTTCAACACATAGAAGATGTTGGTCCAGGATTATGTATGCTTAATATCA
TAGGATTAGATTTTCCAGTACCAACCTTTATTCTAGGTGACCCATTCATGAGAAAATATTTTACCGTCTTTGATTATGAT
AATCAGAGTGTTGGTATTGCTCTTGCTAAAAAGAATTTAGTTATCGGAGCAGGTGCAGGTATGGGAAGTAAAGGAGAA
GAACTTTTCACTGGAGTTGTCCCAATTCTTGTTGAATTAGATGGTGATGTTAATGGGCACAAATTTTCTGTCAGTGGAG
AGGGTGAAGGTGATGCAACATACGGAAAACTTACCCTTAAATTTATTTGCACTACTGGAAAACTACCTGTTCCTTGGC
CAACACTTGTCACTACTTTAACTTATGGTGTTCAATGCTTTTCAAGATACCCAGATCATATGAAACGGCATGACTTTTTC
AAGAGTGCCATGCCCGAAGGTTATGTACAGGAAAGAACTATATTTTTCAAAGATGACGGGAACTACAAGACACGTGCT
GAAGTCAAGTTTGAAGGTGATACCCTTGTTAATAGAATCGAGTTAAAAGGTATTGATTTTAAAGAAGATGGAAACATTC
TTGGACACAAATTGGAATACAACTATAACGATCACCAGGTGTACATCATGGCAGACAAACAAAAGAATGGAATCAAAG
CTAACTTCAAAATTAGACACAACATTGAAGATGGAGGCGTTCAACTAGCAGACCATTATCAACAAAATACTCCAATTGG
CGATGGGCCCGTCCTTTTACCAGACAACCATTACCTGTTTACAACTTCTACTCTTTCGAAAGATCCCAACGAAAAGAG
AGACCACATGGTCCTTCTTGAGTTTGTAACAGCTGCTGGGATTACACATGGCATGGATGAACTATACAAAGATAACTA
AAAACGATTATTAGGATACTAAAGAAAAAACATAATTATATAAGTATAATTATATATATTCACATTTTTTATATATGAATAT
AAGAAAACATATTTTATCCATAATGAACCATTTCATAGAAATTTAAATAATTAAAAAATATTTTTTATTTTTTTTTATTTTTA
TTTTTATTTTTTTATTTTATTTTTTTTTTTTTTTTTATTTTTTTTTTTTACATTCTCCGTATTCTTGTAAAAGAATATAATGATAA
TAAAAACATTTTTTTATTATGATTTTATAATTTTTTATATTTAATTTTTTTTTATTTTTTTCATAGCATTAATTAATATATATATAT
ATATATATATTAGTTATATTTGTGAAATAACTTTTCTTTAATAACCTAACACAAGAGTGAGTTCATAAAATTTTATATTTTG
CCATATATATATATATATATATATATATATATATATATATATATATATATATATACATATATATATTTATGTATATATGTGTTTT
TCCAATATTGGTTCACATTTCTTCTTTTTTCACTCGAAAATATTTGACTTATTATATTTTATATTATATATATATAAAAAAAAA
AAAAAAATTGTTAAAG

Homology regions, linker, SEP

Movie S1 (separate file). Time-lapse microscopy reveals DVM fission upon loss of V-ATPase subunit c. *c cKO* parasites expressing CRT-mNG were tightly synchronized, treated with RAP from 18 hours post invasion onward and imaged live on a confocal microscope throughout intraerythrocytic development. For each timepoint, average CRT-mNG intensity projections from 3D reconstructions are shown alongside a single z-section of DIC. Time stamp, hours:minutes.

Dataset S1 (separate file). Raw fluorescence readings from the drug sensitivity assays depicted in Fig. 5 and *SI Appendix*, Fig. S9.

SI References

1. E. Knuepfer, M. Napiorkowska, C. van Ooij, A. A. Holder, Generating conditional gene knockouts in *Plasmodium* - a toolkit to produce stable DiCre recombinase-expressing parasite lines using CRISPR/Cas9. *Sci Rep* **7**, 3881 (2017).
2. A. Alder *et al.*, A non-reactive natural product precursor of the duocarmycin family has potent and selective antimalarial activity. *Cell Chem Biol* **29**, 840-853 (2021).
3. M. C. Berenbaum, A method for testing for synergy with any number of agents. *J Infect Dis* **137**, 122-130 (1978).
4. F. C. Odds, Synergy, antagonism, and what the chequerboard puts between them. *J Antimicrob Chemother* **52**, 1 (2003).
5. J. M. Matz *et al.*, A lipocalin mediates unidirectional heme biomineralization in malaria parasites. *Proc Natl Acad Sci U S A* **117**, 16546-16556 (2020).
6. C. Grüring *et al.*, Development and host cell modifications of *Plasmodium falciparum* blood stages in four dimensions. *Nat Commun* **2**, 165 (2011).
7. P. Mesén-Ramírez *et al.*, The parasitophorous vacuole nutrient channel is critical for drug access in malaria parasites and modulates the artemisinin resistance fitness cost. *Cell Host Microbe* **29**, 1774-1787 e1779 (2021).
8. K. J. Saliba, H. A. Horner, K. Kirk, Transport and metabolism of the essential vitamin pantothenic acid in human erythrocytes infected with the malaria parasite *Plasmodium falciparum*. *J Biol Chem* **273**, 10190-10195 (1998).
9. E. Knuepfer *et al.*, RON12, a novel *Plasmodium*-specific rhoptry neck protein important for parasite proliferation. *Cell Microbiol* **16**, 657-672 (2014).
10. J. M. Combrinck *et al.*, Insights into the role of heme in the mechanism of action of antimalarials. *ACS Chem Biol* **8**, 133-137 (2013).
